# Matching the Diversity of Sulfated Biomolecules: Creation of a Classification Database for Sulfatases Reflecting Their Substrate Specificity

**DOI:** 10.1371/journal.pone.0164846

**Published:** 2016-10-17

**Authors:** Tristan Barbeyron, Loraine Brillet-Guéguen, Wilfrid Carré, Cathelène Carrière, Christophe Caron, Mirjam Czjzek, Mark Hoebeke, Gurvan Michel

**Affiliations:** 1 Sorbonne Universités, UPMC Univ Paris 06, CNRS, UMR 8227, Integrative Biology of Marine Models, Station Biologique de Roscoff, CS 90074, Roscoff, Bretagne, France; 2 CNRS FR 2424, Sorbonne Universités, UPMC Univ Paris 06, FR2424, ABiMS platform, Station Biologique de Roscoff, CS 90074, Roscoff, Bretagne, France; Weizmann Institute of Science, ISRAEL

## Abstract

Sulfatases cleave sulfate groups from various molecules and constitute a biologically and industrially important group of enzymes. However, the number of sulfatases whose substrate has been characterized is limited in comparison to the huge diversity of sulfated compounds, yielding functional annotations of sulfatases particularly prone to flaws and misinterpretations. In the context of the explosion of genomic data, a classification system allowing a better prediction of substrate specificity and for setting the limit of functional annotations is urgently needed for sulfatases. Here, after an overview on the diversity of sulfated compounds and on the known sulfatases, we propose a classification database, SulfAtlas (http://abims.sb-roscoff.fr/sulfatlas/), based on sequence homology and composed of four families of sulfatases. The formylglycine-dependent sulfatases, which constitute the largest family, are also divided by phylogenetic approach into 73 subfamilies, each subfamily corresponding to either a known specificity or to an uncharacterized substrate. SulfAtlas summarizes information about the different families of sulfatases. Within a family a web page displays the list of its subfamilies (when they exist) and the list of EC numbers. The family or subfamily page shows some descriptors and a table with all the UniProt accession numbers linked to the databases UniProt, ExplorEnz, and PDB.

## Introduction

Widespread in nature, sulfated biomolecules are highly diverse in chemical structure and biological function. These compounds include sulfate esters (ROSO_3_^-^) and sulfamates (RN(H)SO_3_^-^) and range from small molecules to complex polymers. Sulfatases are the key enzymes in the recycling of these compounds, but relatively few sulfatases have been characterized in comparison to the diversity of sulfated biomolecules, and with the explosion of genomic data this gap is increasing. Furthermore, the annotation of sulfatases is prone to errors, notably in term of substrate specificity. After an illustration of the diversity of sulfated compounds found in eukaryotes and microorganisms we will give an overview on the current knowledge on sulfatases, highlighting the need for a classification system for this enzyme class.

Several classes of sulfated compounds have been especially studied in humans and other vertebrates: cerebrosides sulfates, a group of sulfated glycosphingolipids found in nerve cell membranes [1]; steroids sulfates, which serve as precursors for estrogens, androgens and cholesterols [2]; and glycosaminoglycans (GAG) which are major structural constituents of the extracellular matrix and participate in numerous physiological processes [3]. GAG are not unique to vertebrates, but are also widespread in invertebrates [4]. Marine invertebrates synthesize additional extracellular sulfated polysaccharides such as sulfated fucans, mainly found in echinoderms, and sulfated galactans, found in sea squirts (ascidians) and some sea urchin species [5]. Terrestrial plants produce various sulfated secondary metabolites: some key signaling molecules, such as sulfated flavonoids [6] and sulfated derivatives of jasmonic acid [7]; glucosinolates, which are defense metabolites in crucifers [8]; and choline sulfate, which acts as an osmoprotectant in response to salinity or drought stress [9]. All marine macrophytes synthesize sulfated polysaccharides which are major components of their cell wall: sulfated galactans in seagrasses; ulvans and sulfated galactans in green algae; agars, carrageenans and porphyrans in red algae; and sulfated fucoidans in brown algae [10–12]. Extracellular sulfated polysaccharides are also produced by marine unicellular algae, in every studied phylum: green microalgae [13], red microalgae [14], diatoms [15] and haptophytes [16]. Red and brown macroalgae produce a second class of sulfated polymers, phlorotanins, which are sulfated and/or halogenated polyphenols involved in bioadhesion [17]. In prokaryotes the presence of sulfated biomolecules is less systematic and their function depends on species. In rhizobia-legume symbioses the formation of nitrogen-fixing nodules in plant roots is elicited by sulfated chitooligosaccharides called nod factors secreted by bacteria [18]. The sulfation pattern of these nod factors determines the symbiotic host specificity [19]. Mycobacteria produce a complex array of sulfated molecules which modulate host-pathogen interactions [20]. Finally, sulfated exopolysaccharides were characterized in various *Bacteria* and *Archaea* [21, 22]. The above list of sulfated biomolecules is not exhaustive but illustrates the diversity of these compounds, present throughout the tree of life in both terrestrial and marine environments, which play diverse key roles in free-living or symbiotic life styles.

With the sulfotransferases, the sulfatases are the key enzymes in sulfate metabolism. They catalyze the removal of sulfate groups according to either a hydrolytic mechanism (sulfuric ester hydrolases EC 3.1.6.- and sulfamidases EC 3.10.1.-) or an oxidative mechanism (dioxygenase EC 1.14.11.-) [23]. We propose to revise the nomenclature of all sulfatases present in the UniProt databank to improve the accuracy of their functional annotation, creating four families based on sequence similarities, and dividing the family of the formylglycine-dependent sulfohydrolases (FGly-sulfatases) into substrate-specific subfamilies. This classification system is implemented in an online database dedicated to sulfatases, SulfAtlas (http://abims.sb-roscoff.fr/sulfatlas/). Thus, it is possible to distinguish four families of sulfatases: the FGly-sulfatases [24]; the alkylsulfodioxygenases, represented by the alkylsufatase AtsK from *Pseudomonas putida* S-313 [25]; the alkylsulfohydrolases, represented by the alkylsulfatase SdsA1 from *Pseudomonas aeruginosa* PAO1 [26]; and the arylsulfohydrolases, represented by the arylsulfatase AtsA from *Pseudoalteromonas carrageenovora* 9^T^ [27].

**Fig 1:**
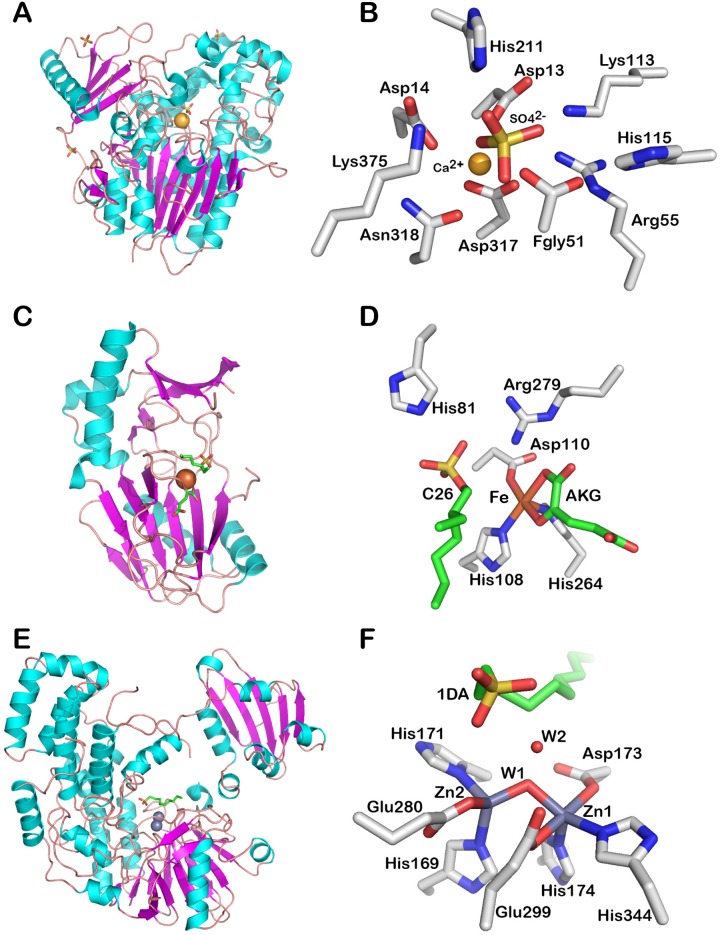
Fold and active site of representatives from the different families of sulfatases. S1 family: Fold (A) and active site (B) of the arylsulfatase AtsA from *Pseudomonas aeruginosa* PAO1 (PDB code: 1HDH) [35]. S2 family: Fold (C) and active site (D) of the alkylsulfatase AtsK from *Pseudomonas putida* S-313 (PDB code: 1OIK) [23]; S3 family: Fold (E) and active site (F) of the alkylsulfatase SdsA1 from *Pseudomonas aeruginosa* PAO1 (PDB code: 2CFU) [65]. The folds are shown in cartoon representation. The amino acids and ligands of the active sites are shown in sticks. The cations are shown as spheres. The figures were made using PyMoL (Version 1.8 Schrödinger, LLC).

**Fig 2:**
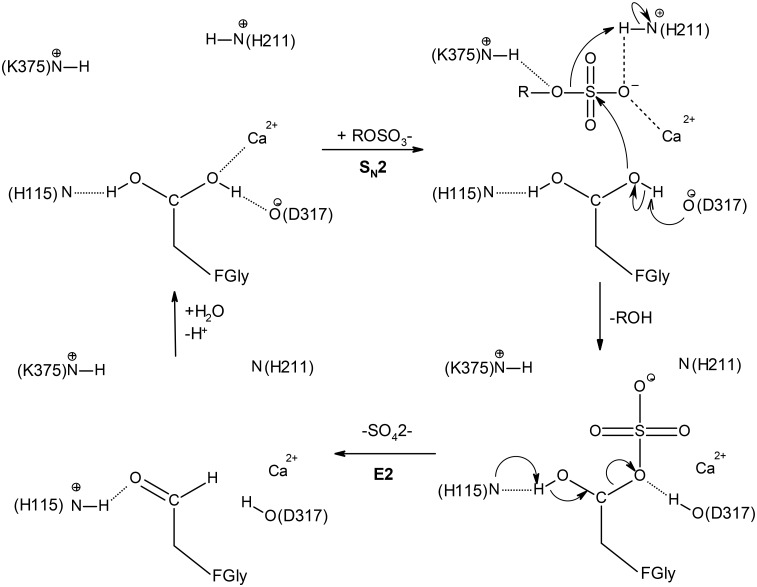
Favored catalytic mechanism of the S1 family sulfatases. The numbering corresponds to the arylsulfatase AtsA from *Pseudomonas aeruginosa* PAO1 [35]. Upon substrate binding, the formyglycine is activated for nucleophilic attack on sulfur by Asp317. The sulfoenzyme intermediate is formed, and desulfation most likely occurs by elimination from the remaining fGly-diol hydroxyl (E2), catalyzed by His115. This figure was adapted from the following references [35, 38] and prepared with Accelrys Draw 4.2.

The vast majority of sulfatases are hydrolytic enzymes containing a unique catalytic residue, the (2S)-2-amino-3-oxopropanoic acid or 3-oxoalanine, also called C_α_-formylglycine (FGly), which is post-translationally generated from a conserved cysteine or serine [28, 29]. The post-translational modification occurs when the polypeptide chain is still unfolded and is directed by a conserved N-terminal [CS]-x-P-x-R motif [30, 31]. Crystal structures have been determined for five human and one bacterial FGly-sulfatases (Table 1) [32–37]. Despite relatively low pair-wise sequence identities (26–34%, Table 2) these proteins adopt a similar fold (Fig 1A) comprising two (α/β) domains consisting of a large N-terminal domain, containing the catalytic pocket (Fig 1B), and a smaller C-terminal domain. Upon substrate binding, the formyglycine is activated for nucleophilic attack on the sulfur by an aspartate (Asp317, AtsA numbering, PDB: 1HDH; Uniprot: P51691). The sulfoenzyme intermediate is formed, and desulfation most likely occurs by elimination from the remaining FGly-diol hydroxyl (E2), catalyzed by a histidine base (His115) (Fig 2) [35, 38]. Thirty-six FGly-sulfatases, mainly from mammals, have been currently characterized at the level of their cDNA, mRNA or gene products and for their substrate specificity (Table 1). However, thirty of these enzymes represent only 9 EC numbers (the six remaining enzymes have not been attributed EC numbers). Most of these enzymes were studied in the context of severe metabolic disorders in man and other mammals. Genetic defects in GAG-specific FGly-sulfatases provoke various mucopolysaccharidoses [39–46], while absence or malfunctioning of cerebroside sulfatase and sterylsulfatase results into metachromatic leukodystrophy and X-linked ichthyosis, respectively [47–49]. However, other FGly-sulfatases have been characterized in various biological and ecological contexts. A herbivorous insect produces a glucosinolate sulfatase which is essential for its resistance to crucifer defense system [50]. Mucin-desulfating sulfatases are secreted by colonic bacteria which degrade mucin glycoproteins in inflammatory conditions of the gastrointestinal tract [51]. The legume symbiont *Ensifer meliloti* synthesizes a choline sulfatase which metabolizes choline-O-sulfate into the osmoprotectant glycine betaine to cope with osmotic stress [52]. Bacterial arylsulfatases are involved in sulfur scavenging from phenolic compounds abundant in soils [53–55]. Additional FGly-sulfatase genes were cloned from human and mouse (ARSD to ARSK) [56–59], from sea urchins [60, 61], from fungi [62] and from green microalgae [63, 64]. But their gene products were only tested on artificial aromatic substrates and their physiological substrates have not been identified yet.

**Table 1:** Sulfatases of known substrate specificity. The proteins have been sorted according to their EC numbers.

Protein name / Family	Gene name	Organism	EC number	UniProt code	PDB code	References
Arylsulfatase / S1_4	*atsA*	*Enterobacter aerogenes* W70	3.1.6.1	P20713	-	[103]
Arylsulfatase / S1_4	*atsA*	*Pseudomonas aeruginosa* PAO1	3.1.6.1	P51691	1hdh	[35, 53]
Arylsulfatase (tyrosine sulfatase) / S1_6		*Volvox carteri*	3.1.6.1	Q10723	-	[64]
Arylsulfatase / S4	*atsA*	*Pseudoalteromonas carrageenovora* 9^T^	3.1.6.1	P28607	-	[27]
Steryl-sulfatase / S1_3	STS (ARSC)	*Homo sapiens*	3.1.6.2	P08842	1p49	[34, 48, 49]
Steryl-sulfatase / S1_3	STS (ARSC)	*Rattus norvegicus*	3.1.6.2	P15589		[104]
Steryl-sulfatase / S1_3	STS (ARSC)	*Mus musculus*	3.1.6.2	P50427		[105]
N-acetylgalactosamine -6-sulfatase / S1_5	GALNS	*Homo sapiens*	3.1.6.4	P34059	4fdi	[36, 40]
N-acetylgalactosamine -6-sulfatase / S1_5	GALNS	*Mus musculus*	3.1.6.4	Q571E4		[106]
N-acetylgalactosamine -6-sulfatase / S1_5	GALNS	*Sus scrofa*	3.1.6.4	Q8WNQ7		[107]
Choline-sulfatase / S1_12	*betC*	*Ensifer meliloti* 1021	3.1.6.6	O69787	-	[52]
Cerebroside sulfatase / S1_1	ARSA	*Homo sapiens*	3.1.6.8	P15289	1auk	[32, 47]
Cerebroside sulfatase / S1_1	ARSA	*Mus musculus*	3.1.6.8	P50428		[108]
N-acetylgalactosamine -4-sulfatase / S1_2	ARSB	*Homo sapiens*	3.1.6.12	P15848	1fsu	[33, 39]
N-acetylgalactosamine -4-sulfatase / S1_2	ARSB	*Felis catus*	3.1.6.12	P33727		[109]
N-acetylgalactosamine -4-sulfatase / S1_2	ARSB	*Rattus norvegicus*	3.1.6.12	P50430		[110]
N-acetylgalactosamine -4-sulfatase / S1_2	ARSB	*Mus musculus*	3.1.6.12	P50429		[111]
Iduronate 2-sulfatase / S1_7	IDS	*Homo sapiens*	3.1.6.13	P22304	-	[112]
Iduronate 2-sulfatase / S1_7	IDS	*Mus musculus*	3.1.6.13	Q08890	-	[42]
Heparin/heparan sulfate 2-O-sulfatase / S1_9	*FH2S*	*Pedobacter heparinus* ATCC 13125^T^	3.1.6.13	C6Y1N2	-	[46]
N-acetylglucosamine-6-sulfatase / S1_6	GNS	*Homo sapiens*	3.1.6.14	P15586	-	[41]
N-acetylglucosamine-6-sulfatase / S1_6	GNS	*Capra hircus*	3.1.6.14	P50426	-	[113]
Mucin-desulfating sulfatase / S1_11	*mdsA*	*Prevotella* sp. RS2	3.1.6.14	Q9L5W0	-	[51]
Extracellular sulfatase 1 (N-acetylglucosamine-6-sulfatase) / S1_6	SULF1	*Coturnix coturnix*	3.1.6.14	Q90XB6	-	[44]
Extracellular sulfatase 2 (N-acetylglucosamine-6-sulfatase) / S1_6	SULF1	*Homo sapiens*	3.1.6.14	Q8IWU6	-	[45]
Extracellular sulfatase 2 (N-acetylglucosamine-6-sulfatase) / S1_6	SULF1	*Mus musculus*	3.1.6.14	Q8K007	-	[45]
Extracellular sulfatase 2 (N-acetylglucosamine-6-sulfatase) / S1_6	SULF2	*Homo sapiens*	3.1.6.14	Q8IWU5	-	[45]
Extracellular sulfatase 2 (N-acetylglucosamine-6-sulfatase) / S1_6	SULF2	*Mus musculus*	3.1.6.14	Q8CFG0	-	[45]
Heparin/heparan sulfate 6-O-sulfatase / S1_11	Phep_2827	*Pedobacter heparinus* ATCC 13125^T^	3.1.6.14	C6Y1N4	-	[114]
Sec-alkysulfatase / S3	*pisA1*	*Pseudomonas* sp. RHO23	3.1.6.19	F8KAY7	2yhe	[115]
N-sulfoglucosamine sulfohydrolase / S1_8	SGSH	*Homo sapiens*	3.10.1.1	P51688	4miv	[37, 43]
Heparin/heparan sulfate N-sulfamidase / S1_8	*Nsulf*	*Pedobacter heparinus* ATCC 13125^T^	3.10.1.1	C6Y1N3	-	[116]
Alkysulfatase / S2	*atsK*	*Pseudomonas putida* S-313	1.14.11.-	Q9WWU5	1oih	[23, 25]
Alpha-ketoglutarate-dependent sulfate ester dioxygenase / S2	*Rv3406*	*Mycobacterium tuberculosis* H37Rv^T^	1.14.11.-	P9WKZ1	4cvy	[117]
Endo-4S-kappa-carrageenan sulfatase / S1_7	Patl_0891	*Pseudoalteromonas atlantica* T6c	3.1.6.-	Q15XH1		[93]
Glucosinolate sulfatase / S1_10	-	*Plutella xylostella*	3.1.6.-	Q8MM72	-	[50]
Endo-4S-iota-carrageenan sulfatase / S1_19	Patl_0889	*Pseudoalteromonas atlantica* T6c	3.1.6.-	Q15XH3		[92]
Endo-4S-kappa-carrageenan sulfatase / S1_19	Patl_0895	*Pseudoalteromonas atlantica* T6c	3.1.6.-	Q15XG7		[93]
Alkysulfatase / S3	*sdsA1*	*Pseudomonas aeruginosa* PAO1	3.1.6.-	Q9I5I9	2cfu	[26, 65]
Alkysulfatase / S3	*psdsA*	*Pseudomonas* sp. S9	3.1.6.-	F2WP51	4nur	unpublished
phosphonate monoester hydrolase / phosphodiesterase / S1_0		*Burkholderia caryophylli* PG2982	3.1.-.-	Q45087	2w8s	[118]
phosphonate monoester hydrolase / phosphodiesterase / S1_0		*Rhizobium leguminosarum bv*. *viciae* 3841	3.1.-.-	Q1M964	2vqr	[98]

**Table 2:** Identity scores for pairwise sequence comparisons of the formyglycine-dependent sulfatases of known substrate specificity. For each entry, the bold numbers correspond to the identity score for full length sequences, while the numbers in italics correspond to the identity score after editing of the multiple sequence alignment.

	ARSA	ARSB	ARSC	AtsAp	AtsAk	GALNS	GNS	SULF2	SULF1	IDSh	SGSH	ID2Sp	GlcS	MdSA	betC
ARSA	100	**27.9** *30*.*5*	**33.3** *36*.*9*	**25.8** *30*.*7*	**25.4** *26*.*0*	**36.5** *41*.*3*	**23.9** *23*.*6*	**17.6** *22*.*4*	**16.6** *22*.*0*	**23.9** *26*.*7*	**27.2** *27*.*9*	**23.5** *25*.*3*	**25.3** *26*.*0*	**25.3** *27*.*5*	**25.3** *27*.*9*
ARSB		100	**25.7** *30*.*0*	**23.8** *30*.*5*	**23.0** *27*.*8*	**29.1** *31*.*7*	**22.2***24*.*3*	**16.9** *21*.*2*	**16.9** *22*.*1*	**22.6** *24*.*0*	**22.6** *26*.*7*	**22.9** *25*.*5*	**32.0** *36*.*0*	**21.9** *25*.*5*	**20.1** *22*.*4*
ARSC			100	**24.0** *26*.*5*	**22.4** *24*.*0*	**31.5** *36*.*4*	**21.5** *22*.*8*	**17.6** *23*.*8*	**15.0** *22*.*6*	**23.1** *26*.*2*	**23.3** *25*.*2*	**22.4** *26*.*0*	**22.4** *25*.*9*	**22.6** *26*.*5*	**23.9** *26*.*4*
AtsAp				100	**35.3** *40*.*0*	**25.1** *28*.*6*	**19.8** *23*.*6*	**17.4** *20*.*4*	**16.4** *20*.*6*	**20.2** *22*.*4*	**25.2** *28*.*7*	**21.4** *22*.*3*	**25.4** *29*.*3*	**25.0** *28*.*0*	**24.5** *26*.*3*
AtsAk					100	**22.6** *24*.*2*	**18.0** *21*.*1*	**17.0** *19*.*8*	**16.5** *19*.*4*	**20.1** *21*.*5*	**21.2** *21*.*3*	**21.1** *21*.*9*	**25.0** *28*.*9*	**22.9** *25*.*4*	**23.7** *26*.*0*
GALNS						100	**21.3** 23.3	**16.7** *24*.*2*	**16.4** *21*.*6*	**23.6** *26*.*4*	**27.0** *29*.*4*	**23.3** *24*.*3*	**25.6** *26*.*2*	**22.4** *25*.*6*	**21.1** *24*.*5*
GNS							100	**26.5** *41*.*5*	**25.3** *39*.*9*	**21.3** *21*.*6*	**24.0** *24*.*3*	**21.7** *22*.*2*	**20.5** *20*.*7*	**25.0** *29*.*2*	**21.9** *23*.*0*
SULF2								100	**64.1** *81*.*0*	**16.2** *21*.*5*	**16.7** *21*.*0*	**15.2** *24*.*0*	**17.2** *22*.*1*	**17.0** *27*.*6*	**15.1** *22*.*0*
SULF1									100	**15.7** *20*.*4*	**16.3** *23*.*2*	**15.7** *22*.*8*	**17.8** *23*.*8*	**17.3** *27*.*8*	**16.0** *21*.*5*
IDSm										100	**21.8** *25*.*1*	**22.2** *22*.*3*	**20.3** *21*.*3*	**21.7** *23*.*9*	**26.5** *30*.*1*
SGSH											100	**21.9** *22*.*0*	**23.1** *23*.*3*	**24.4** *25*.*9*	**22.7** *24*.*0*
ID2Sp												100	**21.8** *24*.*0*	**24.4** *26*.*4*	**23.1** *26*.*1*
GlcS													100	**23.2** *24*.*6*	**22.4** *22*.*6*
MdsA														100	**23.2** *26*.*2*
BetC															100

**Fig 3:**
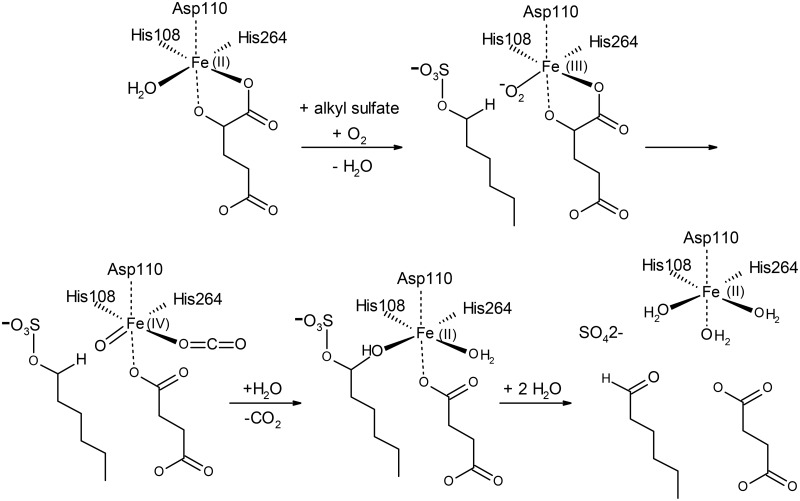
Catalytic mechanism of the S2 family sulfatases. The numbering corresponds to the alkylsulfatase AtsK from *Pseudomonas putida* S-313. First iron and the cosubstrate alpha-ketoglutarate (KG) coordinate to the enzyme. Second, the alkyl sulfate binds to the active site, displacing a water molecule from the iron center and liberating an unsaturated iron atom. Subsequently a dioxygen molecule binds the iron cation. One oxygen atom of the dioxygen is transferred to KG, yielding succinate and carbon dioxide as products. The iron is thereby oxidized, and a ferryl Fe(IV) = O species is formed, which then hydroxylates the alkyl sulfate via a radical intermediate. Finaly sulfate ion and succinate are released and two water molecules complete the iron coordination sphere. This figure was adapted from [23, 119, 120]

The three other families of sulfatases are rather small in comparison to the FGly-sulfatases. The alkylsufatase AtsK from *P*. *putida* S-313 is a dioxygenase which, in presence of Fe(II) as cofactor, converts one molecule of α-ketoglutaric acid (αKG) and one molecule of dioxygen, used as co-substrates, into succinic acid and carbon dioxide per molecule of cleaved sulfate ester (Fig 3) [25]. The crystal structure of this enzyme reveals a jellyroll fold similar to the other known Fe αKG-dependent dioxygenases (Fig 1C and 1D) [23]. The alkylsulfatase SdsA1 from *P*. *aeruginosa* PAO1 is a hydrolase featuring an N-terminal catalytic domain, a central dimerization domain and a C-terminal hydrophobic domain recruiting aliphatic substrates. The catalytic domain of SdsA1 adopts a metallo-β-lactamase fold (Fig 1E) and binds two zinc ions as cofactors (Fig 1F) [26, 65]. Nonetheless, its catalytic mechanism remains ambiguous [65]. Another sulfate hydrolase, the arylsulfatase AtsA from *P*. *carrageenovora* 9^T^ [27], also possesses the conserved histidines forming the zinc-binding motif of the metallo-β-lactamase superfamily [66]; however, AtsA does not display other significant sequence similarity with the catalytic domain of the alkylsulfatase SdsA1 (~13% sequence identity). Altogether, the number of characterized sulfatases remains limited and does not reflect the huge chemical diversity of the sulfated biomolecules.

With the genomic revolution the number of sulfatase sequences is constantly increasing. For instance, the genome sequencing of the marine planctomycete *Rhodopirellula baltica* SH1^T^ has been an exceptional event in the field of sulfatases. Indeed this bacterium contains the largest number of FGly-sulfatases to date (104 genes) [67] and this trend has been confirmed in other species of this genus [68]. Large numbers of sulfatases have been also identified in marine flavobacteria known to degrade sulfated polysaccharides from seaweeds, such as *Formosa agariphila* (49 FGly-sulfatases) [69] and *Zobellia galactanivorans* (71 FGly-sulfatases) (Barbeyron et al., Environmental Microbiology, in revision). In the terrestrial environment, the genome of the GAG-degrading sphingobacterium *Pedobacter heparinus* is also rich in sulfatases with 20 FGly-sulfatases. Such new sulfatases originating from genomic data are most often simply annotated as “sulfatases” or “arylsulfatases”, which is not precise enough to predict the metabolic pathways in which these enzymes are involved. In less studied organisms or ecosystems, an annotation only based on the similarity with the currently characterized sulfatases (Table 1) is likely to incorrectly predict substrate specificity.

In order to improve the predicted sulfatase substrate specificities we have undertaken an extensive census of the sulfatase sequences available in the Uniprot database. Multiple alignments were calculated in order to determine or update the consensus sequences conserved in each family of sulfatases. These alignments were also used for phylogenetic analyses. Notably in the family of FGly-sulfatases the sequences diverge into 73 clades that coincide with their substrate selectivity. Most of the clades do not encompass characterized FGly-sulfatases, supporting the existence of subfamilies of FGly-sulfatases with novel unidentified substrate specificities.

## Materials and Methods

Sulfatase sequences were extracted from the UniProt database in August 2009 using the BlastP program [70]. Alkylsulfohydrolases (370 proteins) and arylsulfohydrolases (15 proteins), which belong to the metallo-β-lactamase superfamily, were identified by at least 30% sequence identity over ~600 residues with the characterized enzymes alkylsulfatase SdsA1 (Uniprot code: Q9I5I9) and arylsulfatase AtsA (P28607), respectively, and by the presence of the pattern HxHxDH, which is involved in the coordination of two catalytic zinc ions. Fe αKG-dependent alkylsulfodioxygenases (111 proteins) were identified by at least 30% sequence identity over ~300 residues with the characterized alkylsulfodioxygenase AtsK (Q9WWU5) and by the presence of the pattern HxD/Ex_n_H (n = 39 to 154) involved in the coordination of the Fe ion [23]. The extracted sulfatase sequences were subjected to multiple sequence alignments using the MAFFT [71] program, with the iterative refinement method L-INS-i and the scoring matrix Blosum62. Complete sets of orthologous alkysulfohydrolases and arylsulfohydrolases on one hand, and alkylsulfodioxygenases on the other hand, were classified based on phylogenetic analyzes using the metallo-β-lactamases and Fe αKG-dependent dioxygenase superfamilies, respectively.

The identification of FGly-sulfatases (4058 proteins) was based on a significant level of sequence identity of at least 25% with characterized enzymes (Table 1) over a minimal length compatible with the size of the known FGly-sulfatases (at least 400 residues), and by the conservation of the two PROSITE signatures PS00523 and PS00149 which correspond to the simplified patterns [SAPG]-[LIVMST]-[CS]-[STACG]-P-[STA]-R-x(2)-[LIVMFW](2)-[TAR]-G and G-[YV]-x-[ST]-x(2)-[IVAS]-G-K-x(0,1)-[FYWMK]-[HL], respectively [30, 31]. The proteins encompassing several FGly-sulfatase modules were divided into distinct sequences corresponding to each catalytic module. Due to the huge number of sequences, it is impossible to directly obtain a reliable multiple alignment of this large group of sequences. Therefore, the FGly-sulfatase sequences were first divided into 81 groups and 32 orphan sequences, on the basis of sequence identities using the BlastP program. A multiple sequence alignment was obtained for each of these groups using MAFFT [71] with the iterative refinement method L-INS-i and the scoring matrix Blosum62. Then these 81 multiple sequence alignments were manually stacked on each other by matching similar zones using Jalview [72]. The alignments were manually improved using Jalview on the basis of the sequence alignment derived from the superposition of available crystal structures of sulfatases (Table 1). After this refinement step, the poorly conserved regions were removed from the multiple sequence alignment. The different phylogenetic trees were derived from these refined alignments using Maximum Likelihood method with the program RAxML with the MTMAMF or WAG as substitution matrix [73] or with the program MEGA 5.2.2 [74]. The reliability of the trees was always tested by bootstrap analysis using 100 resamplings of the dataset. The trees were displayed with MEGA 5.2.2 [74]. For the FGly-sulfatase sequences, the program MatGat [75] was used and two identity matrices were generated, one for the full length proteins and the second matrix corresponding to the edited multiple sequence alignment. The logo sequences were built using WebLogo via the PROSITE databank [76].

## Results

### Analyses of alignment of formylglycine-dependent sulfatases (family S1)

**Fig 4:**
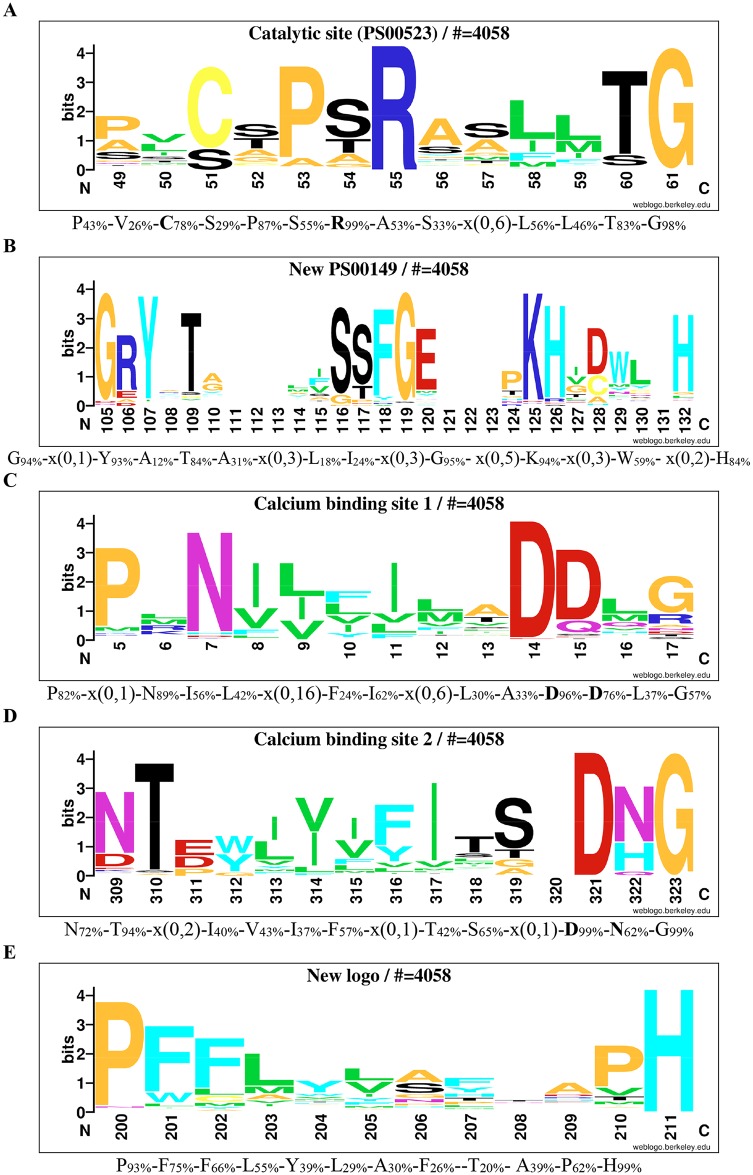
Logos of conserved consensus sequences identified in the global alignment of FGly-sulfatases. Logos of conserved consensus sequences were identified from 4058 aligned FGly-sulfatases. The logo sequence of the catalytic site that corresponds to the PROSITE signature PS00523, is shown in A. The logo sequence of PROSITE signature PS00149 is shown in B. The two logo sequences of calcium binding are shown in C and D. A logo sequence from a conserved supplementary consensus sequences is shown in E. The numbers below the logo sequences indicate, at the first position, the corresponding position in reference sequence (AtsA P51691). The corresponding consensus sequences in multi-alignment are shown below the logo sequences. The percentages in subscript are the percentages of sequences, where the amino acid is conserved in alignment. Catalytic amino acids and residues involved in calcium ion binding are in bold.

From 211 FGly-sulfatase sequences used as seed (104 sequences from *R*. *Baltica* SH1^T^, 71 sequences from *Z*. *galactanivorans* Dsij^T^ and the 36 FGly-sulfatases with a known substrate specificities; Table 1), 4058 FGly-sulfatases were extracted from the UniProt database (August 2009). The FGly-sulfatases belongs to the alkaline phosphatase superfamily. They are easily identified using tools such as PFAM or PROSITE which propose the signatures PF00884 (sulfatase) or PS00523 and PS00149. However, these signatures were defined on a limited number of seed sequences (57 for PF00884, 58 for PS00523 and 50 for PS00149) and our multi-alignment shows that these signatures are no longer completely correct. Therefore, we have updated the two signatures, PS00523 and PS00149 (Fig 4A and 4B). Moreover, we have identified three additional conserved signatures, which can be modelled according to PROSITE syntax and illustrated by sequence logos (Fig 4C–4E).

#### Updating of the PROSITE signatures

The PROSITE database describes the consensus pattern PS00523 for the catalytic site. This signature contains the two essential amino acids Cys51 and Arg55 (numbering of the sulfatase AtsA from *Pseudomonas aeruginosa* PAOI as reference, P51691). Cys51 is post-translationally modified to FGly and plays the role of catalytic nucleophile (Fig 1B). Arg55 is involved in the stabilization of FGly residue (Fig 1B). From the 4058 aligned sulfatase sequences, the catalytic site is identified as the consensus signature P_43_-V_26_-**C**_78_-S_29_-P_87_-S_55_-**R**_99_-A_53_-S_33_-x(0,6)-L_56_-L_46_-T_83_-G_98_ (subscript numbers indicate the percentage of conservation in alignment; catalytic amino acids are in bold; S1A Fig). The catalytic nucleophile is a cysteine in 3202 sequences (78.9% of sequences) or a serine in 857 sequences (21.1% of sequences; S1A Fig). The Cys-containing sulfatases originate from eukaryotic and prokaryotic organisms. All the Ser-containing sulfatases are only present in facultative or strictly anaerobic prokaryotes and excluded from strictly aerobic prokaryotes except the sequences B7PTL2 and Q3V1R8 from the eukaryotes *Iodes scapularis* and *Mus musculus*, respectively. The second important catalytic amino acid is Arg55. As expected this residue shows 99% of conservation suggesting that a positively charged residue at this position is crucial for the catalysis. From the final multi-alignment only eleven sequences possess a different amino acid at this position. A lysine and a glutamine are found at this position in the fungal sulfatases B8MGN1 from *Talaromyces stipitatus* 5217.10^T^ and A5AB99 from *Aspergillus niger* CBS 513.88, respectively. Finally, nine sequences belonging to the phyla *Lentisphaerae* and *Planctomycetes* have lost the positively charged residue which is replaced by an isoleucine or a leucine (S1A Fig), suggesting that these putative sulfatases may be inactive. Located between the two catalytic amino acids, Pro53 is conserved in 87% of sequences (S1A Fig). This residue is mainly replaced by alanine (in 369 sequences), the other amino acids each represent less than 1% (S1A Fig). The terminal dipeptide Thr60-Gly61 is also well conserved in the catalytic site signature. Thr60 is conserved in 83% of sequences (S1A Fig) and is replaced by serine in only 480 sequences. Other amino acid substitutions are found only in very few sequences. Gly61 is nearly strictly conserved (98% of aligned sequences; S1A Fig); this residue is structurally important, since it allows the change of direction of the polypeptide chain after the α-helix encompassing the catalytic signature [32]. Nonetheless, this glycine is replaced by other small residues, a serine in 30 sequences or an alanine in 14 sequences. Finally, the insertion "x(0,6)" is due to the sequence A9UYU7 from the Choanoflagellida *Monosiga brevicollis*. The insertion "x(0,6)" was removed to generate the sequence logo shown in Fig 4A. On the model of the PROSITE consensus pattern PS00523, we have updated this pattern, called the catalytic site pattern (Fig 4A), as [SAPG]-[LIVMSTPAR]-[CS]-[STACGMV]-[PA]-[STAGF]-R-x-{PRFWYH}-[LIVMFWYHQ](2)-[TASL]-G. This new catalytic consensus pattern recovered 9339 sequences from TREMBL database (July 2016), including 8949 true FGly-sulfatases (96%).

From our global alignment, the consensus sequence corresponding to the second PROSITE signature (PS00149) is G_94_-x(0,1)-Y_93_-A_12_-T_84_-x(0,42)-A_31_-x(0,3)-L_18_-x(0,13)-I_24_-x(0,3)-G_95_-x(0,5)-K_94_-x(0,3)-W_59_-x(0,2)- H_81_ (S1B Fig). The most conserved amino acids are Gly105, Tyr106, Gly112 and Lys113 (numbering of the sulfatase AtsA from *P*. *aeruginosa* PAOI as reference, P51691). Gly105 is conserved in 94% of sequences (S1B Fig) and is mainly replaced by an aspartic acid or asparagine in 94 and 66 sequences, respectively. Tyr106 is conserved in 93% of sequences (S1B Fig). It is mainly replaced by an isoleucine, present in 98 sequences. Gly112 is conserved in 95% of sequences (S1B Fig). This amino acid is substituted by a serine in 87 sequences. Among the 4058 sequences of FGly-sulfatases, Lys113 is conserved in 94% of sequences (S1B Fig). This residue can be conservatively replaced by an aspartic acid in 87 sequences or an arginine in 61 sequences.

With the exception of the four residues mentioned above (Gly105, Tyr106, Gly112 and Lys113), the signature PS00149 is poorly conserved and presents many insertions between some residues (S1B Fig). Between the residues Gly105 and Tyr106, the "x(0,1)" position is due to 18 sequences, 14 of which are from various species of *Drosophila* that display an arginine at this position. The "x(0,42)" position is due to an insertion of 42 and 35 amino acids provided by the sequences A7SK50 from the anemone *Nematostella vectensis* and Q4SR77 from the fish *Tetraodon nigroviridis*, respectively. At the first "x(0,3)" position, an insertion of 1 to 3 amino acids is present in sequences A6DPE8 and A6DPF2 from *Lentisphaera araneosa* HTCC2155^T^ and in *Planctomycetes* sequences A6C8W8 and D2R663 from *Planctomyces maris* 534-30^T^ and *Pirellula staleyi* Michigan^T^. The "x(0,13)" position is due to ten sequences. The second position "x(0,3)" is present in fifty two sequences. Between the highly conserved residues Gly112 and Lys113 (position "x(0,5)"), an insertion of 1 to 5 residues is provided by more than sixty sequences. The last position "x(0,3)" is due to 334 sequences. Finally, the position "x(0,2)" concerns 91 sequences. To have a global view of this region, we have made a logo sequence with all variable positions, except the "x(0,42)" and "x(0,13)" positions which only involve a dozen sequences (Fig 4B). The "x(0,1)" position, the first "x(0,3)"position and the "x(0,5)" and "x(0,2)" positions were also excluded, in order to build a new consensus pattern not too degenerated in comparison to PS00149. Moreover only residues that represent more than 1% in a conserved position in the 4058 sequences are included in the consensus pattern. The resulting consensus pattern is G-Y-x-[TSCV]-x(3)-G-K-[IVGTLSEHDCA](0,3)-[WMYNLF]-[HLGN]. With this pattern we have recovered 9041 sequences from trEMBL (July 2016) composed of 80% of FGly-sulfatases.

#### Additional conserved signatures

The FGly-sulfatases are calcium-dependent enzymes [24]. Four residues, Asp13, Asp14, Asp317 and Asn318 coordinate the calcium ion (numbering of the sulfatase AtsA from *P*. *aeruginosa* PAOI as reference, Fig 1B). In the final multi-alignment, Asp13 and Asp14 can be included in the conserved sequence P_82_-x(0,1)-N_89_-I_56_-L_42_-x(0,16)-F_24_-I_62_-x(0,6)-L_30_-A_33_-D_**96**_-D_**76**_-L_37_-G_57_ (S1C Fig; amino acids involved in coordination of calcium are in bold). Asp13 is conserved in 96% of sequences. However, in some rare sequences, glutamate, histidine, glycine, asparagine or arginine (S1C Fig) are found at the place of this residue. In contrast Asp14 is less conserved (76% of conservation). The multi-alignment shows that this residue can be replaced by a large number of amino acids (S1C Fig). The insertions "x(0,16)" and "x(0,6)" are due to the sequences B3T1C6 from the uncultured marine microorganism HF4000_009G21 and A8HPB7 from *Chlamydomonas reinhardtii*, respectively. These two sequences have been excluded in order to build a conserved signature useful to identify the FGly-sulfatases. Thus, we propose the following consensus pattern, referred to as Ca-binding 1 pattern (Fig 4C), [PM]-x(0,1)-[NHD]-[IVFL]-[LIV]-[FLVIY]-[IVLF]-[LMVFITYW]-[ATVSLI]-[DE]-[DQ]-[LMQVH]-[GRANTDS]. The corresponding sequence logo is shown in Fig 4C. This consensus pattern was used to query the TREMBL database via the PROSITE website and recovered 9355 sequences (July 2016), mainly annotated sulfatases or arylsulfatases, type I phosphodiesterase/nucleotide pyrophosphatase family protein or uncharacterized protein. Among these sequences, 145 (1.55%) were identified as false positive sequences and 9210 (98.45%) were true FGly-sulfatases. These results suggest that this new consensus pattern will be useful to recover sequences of putative sulfatases in order to assist in the updating of a dedicated database to sulfatases.

The residues Asp317 and Asn318 are also involved in calcium ion coordination (Fig 1B). They are included (in bold) in the conserved signature N_72_-T_94_-x(0,2)-I_40_-V_43_-I_37_-F_57_-x(0,1)-T_42_-S_65_-x(0,1)-D_**99**_-N_**61**_-G_99_ (S1D Fig). The amino acid Asp317, conserved at 99% (S1D Fig), is most frequently replaced by a glutamate in 19 sequences only. Also, some rare amino acids can replace it as threonine, alanine, arginine and tyrosine (S1D Fig). Surprisingly, Asn318 is poorly conserved (61%), although this residue is involved in the calcium coordination and the activation of the FGly residue. While histidine and glutamine are the most frequent residues found in its place, many other amino acids are encountered concerning less than 1% of the sequences each (S1D Fig). Two highly conserved residues, Thr310 (94% of sequences) and Gly319 (99% of sequences), are present in this motif (S1D Fig), although they are not involved in calcium ion binding. Thus we have defined a second consensus signature, called Ca-binding 2 pattern (Fig 4D), [ND]-[TSA]-x(0,2)-[ILVYMF]-[VILF]-[IVFLM]-[FYVL]-x(0,1)-[TSLMIVFAWGC]-[STGA]-D-[NHQ]-G. The position x(0,2) is due to only seven sequences from *Coraliomargarita akajimensis* 04OKA010-24^T^, the sequence F4AN26 from *Paraglaciecola agarilytica* 4H-3-7+YE-5 and the sequence C0FVD6 from *Roseburia inulinivorans* A2-194^T^. The first position "x(0,1)" is due to the same sequences (except C0FVD6) and to 179 sequences which display this supplementary amino acid. The sequence logo corresponding to this consensus pattern is shown in Fig 4D. From interrogation of TREMBL database using the Ca-binding 2 consensus pattern, we have obtained 9299 sequences that included only 7525 sulfatases (81%), a lower efficiency than the Ca-binding 1 consensus pattern.

An additional consensus sequence is P_93_-F_75_-F_66_-L_55_-x(0,1)-Y_39_-x(0,34)-L_29_-A_30_-x(0,1)-F_26_-T_20_-x(0,5)-A_39_-P_62_-H_99_ (S1E Fig). This motif corresponds to the sequence PFFAYLPFSAPH in the reference sequence P51691. Pro200 and His211 are conserved in 93 and 99% of sequences respectively (S1E Fig) suggesting that these amino acids are essential for FGly-sulfatases. Pro200 is structurally important, facilitating the direction change between the α-helix D and the β-strand 10, while His211 is located in the active site (Fig 1B). Pro200 can be replaced by asparagine (2% of sequences) or lysine (1% of sequences). Other amino acids are present at this position, but they represent less than 1% of the sequences each, (S1E Fig). His211 is mainly replaced by a lysine (in ten sequences), the other amino acids concern less than 1% of the sequences each (S1E Fig). Moreover, a small number of sequences provoke some size-variable insertions in the consensus sequence. The first position "x(0,1)" is due to four sequences of which D5EPW8 from *C*. *akajimensis* 04OKA010-24^T^ is also responsible for the insertion at the second position "x(0,1)". The positions "x(0,34)" and "x(0,5)" are due to the sequences B2AAG4 from *Podospora anserina* strain S and A9VAR3 from *M*. *brevicollis*, respectively. After removing of these six sequences, we have defined the consensus pattern P-[FWLI]-[FLCMY]-[LMAVI]-[YWVMTF]-[LVIYFM]-[ASGNP]-{RK}-x(2)-[PVTM]-H that allows recovery of 8359 sequences including 7572 FGly-sulfatases (90,6%) from TREMBL. The corresponding sequence logo is shown in Fig 4E.

From the global alignment, other highly conserved amino acids were found. This is the case for the amino acids Asp291 (98% of conservation) (numbering of the sulfatase AtsA *from P*. *aeruginosa* PAOI as reference, P51691), Lys375 (96%), Asp409 (98%), Thr413 (91%), Gly437 (91%) and Asp495 (95%). Based on the inspection of the crystal structure of the sulfatase AtsA from *P*. *aeruginosa* PAOI (PDB: 1HDH), Asp291, Asp409, Thr413, Gly437 and Asp495 are likely crucial for protein folding. In contrast, Lys375 is localized in the active site (Fig 1B) and is known to be functionally important [35]. However, they are found in very short consensus sequences or associated with many poorly conserved residues and thus can not be used to build a FGly-sulfatase specific consensus pattern.

### Phylogenetic analyses of formylglycine-dependent sulfatases (family S1)

The final multi-alignment (4058 sequences) was manually edited to remove the truncated sequences and all parts of the sequences that were not aligned. The resulting alignment contained 4005 sequences and 329 positions and was used for the phylogenetic studies. Thus, phylogenetic trees were derived using various reconstruction methods. All these methods yielded similar tree topologies, but the maximum-likelihood method using RaxML [73] with the substitution matrices MTMAMF or WAG resulted in the highest bootstrap values and was preferentially chosen (S2 Fig). The differences between the two evolutionary models concerned the bootstrap values where some nodes showed higher bootstrap value with the WAG model, whereas the other bootstrap values were generally higher with the MTMAMF model. On the basis of the substrate specificity, when it is known, and of the deepest nodes in the tree (those nearest to the outgroup) with the highest bootstrap values, 73 clades were identified (S2 Fig). The twelve first clades contained at least one sequence where the substrate specificity was biochemically demonstrated, the other clades represent unknown substrate specificities. Among the clades with known substrate specificity, the activities of cerebroside-sulfatase (EC 3.1.6.8), *N*-acetylgalactosamine-4-sulfatase (EC 3.1.6.12), steryl-sulfatase (EC 3.1.6.2), Arylsulfatase (EC 3.1.6.1), 4*N*-acetylgalactosamine-6-sulfatase (EC 3.1.6.4), *N*-sulfoglucosamine sulfohydrolase (EC 3.10.1.1) and choline-sulfatase (EC 3.1.6.6) are represented by the clades 1, 2, 3, 4, 5, 8 and 12, respectively (S2 Fig). However, two activities are present in more than one clade. The activity iduronate-2-sulfatase (EC 3.1.6.13) is present in the clades 7 and 9. Both clades include prokaryotic sulfatases, but only clade 7 possesses eukaryotic sulfatases. Similarly, the activity N-acetylglucosamine-6-sulfatase (EC 3.1.6.14) is present in clades 6 and 11. The clade with the largest number of sequences (650 sequences) is clade 4 (S2 Fig). All clades are supported by bootstrap values above 65%. Half of the clades have bootstrap values of 99 or 100%, there are two exceptions: clades 14 (82 sequences) and 19 (53 sequences) (S2 Fig), each composed of sequences recovered by BLAST and whose pairwise sequence similarities are about 35%. Although supported by very low bootstrap values (S2 Fig), these two clades are present in all phylogenetic trees tested. Probably, these clades correspond to multiple substrate specificities. Finally, the phylogenetic tree displays 32 orphan sequences spread throughout the tree. Due to their insignificant bootstrap values their position varies within the different trees obtained. It was not possible to include them in the neighboring clades.

### Analyses of alignments and phylogenetic trees of sulfatases belonging to the Fe(II) alpha-ketoglutarate-dependent dioxygenase superfamily (family S2)

The first sulfatase acting with a dioxygenase activity was represented by the alkylsufatase AtsK from *Pseudomonas putida* S-313 [25]. This enzyme was used as query sequence (accession number Q9WWU5) with the algorithm BLASTP to detect the other alkylsulfodioxygenases present in the UniProt database. AtsK displays some similarities with proteins annotated as taurine dioxygenase-related proteins (TauD) and with 2,4-dicholorophenoxyacetate dioxygenase-related proteins (TfdA). An alignment of 469 proteins belonging to the dioxygenase superfamily was realized. A characteristic sequence of the dioxygenase superfamily is the presence of the signature HxD(E)x_n_H (where n is a number comprised between 39 to 154). This signature contains the residues His108, Asp110 and His264 that are involved in the coordination of the Fe ion (numbering of the *P*. *putida* alkylsulfatase AtsK Q9WWU5 as reference; Fig 1D) [23]. The multi-alignment reveals that the residues involved in the coordination of the Fe ion are included, on one hand in the consensus sequence W_96_-**H**_99_-T_71_-**D**_99_-V_66_-T_68_-F_60_ and, on the other hand in the consensus sequence Q_56_-**H**_100_-Y_51_-A_89_-V_29_-A_25_ (subscript numbers indicate the percentage of conservation in dioxygenase alignment and amino acids involved in coordination of Fe are represented in bold). The co-substrate alpha-ketoglutaric acid is coordinated by the Fe ion and by the amino acids Thr135, Arg275 and Arg279 (Fig 1D) [23]. These residues are conserved in the two consensus sequences G_98_-G_99_-D_86_-**T**_100_ and **R**_98_-V_28_-M_39_-H_37_-**R**_98_ (amino acids involved in co-substrate coordination are in bold). In the catalytic site, the sulfate group of the substrate is recognized by the residues His81, Val111 (included in the dioxygenases signature) and Arg279 (Fig 1D) [23]. His81 is conserved in 83% of sequences of the alignment whereas Val111 is only conserved in 66% of sequences.

**Fig 5:**
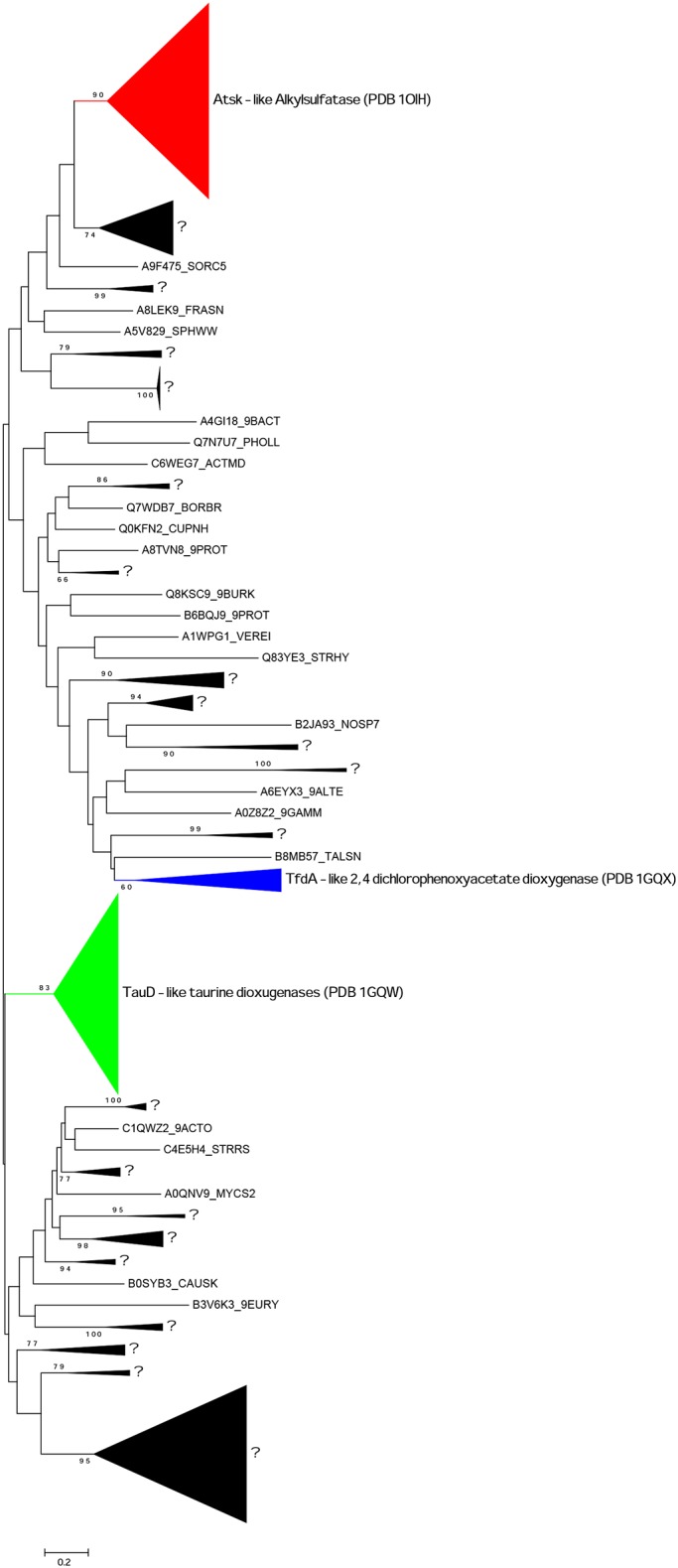
Phylogenetic tree of Fe(II) alpha-ketoglutarate-dependent dioxygenase superfamily. The tree was obtained by maximum likelihood with RAxML using the substitution matrix WAG from 211 positions of an alignment of 469 sequences belonging to the alpha-ketoglutarate-dependent dioxygenase superfamily, closely related to TauD, TfdA and AtsK families. The clades in colors contain the characterized sequences TauD (taurine dioxygenase, P37610), TfdA (2,4-dichlorophenoxyacetate dioxygenase, P10088) and AtsK (alkysulfatase, Q9WWU5). The black clades and the isolated sequences (not supported by high bootstrap values) contain no biochemically-characterized enzymes. The families S2 of the sulfatases is shown in red. All the resolved tridimensional structures are indicated. Only bootstrap values above 60% are shown.

**Fig 6:**
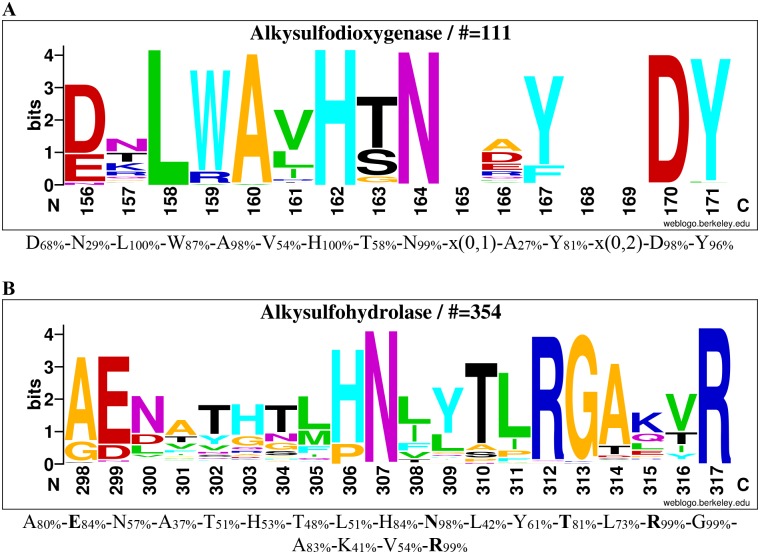
Logos of conserved consensus sequences identified in the global alignment of alkylsulfodioxygenases (family S2) and alkylsulfohydrolases (family S3). Logos sequences identified from aligned 111 alkylsulfodioxygenases (A) and 354 alkylsulfohydrolases (B). The numbers below the logo at the first position indicate the corresponding position in reference sequences (Atsk Q9WWU5 in A and SdsA1 Q9I5I9 in B). The corresponding consensus sequences in multi-alignments are shown below the logo sequences. The percentages in subscript are the percentages of sequences where the amino acid is conserved in alignments. Amino acids involved in sulfate binding are in bold.

Phylogenetic trees were obtained after editing of the multi-alignment to remove the unaligned motifs. All algorithms showed that AtsK was included in a clade composed of 111 sequences with a bootstrap value always above 85% (Fig 5). The proteins TauD (P37610) [77] and TfdA (P10088) [78] each belong to different clades localized elsewhere in the tree (Fig 5). From the alignment of the 111 putative alkylsulfodioxygenases, we observe that the conservation of His81, Val111 and Arg279 (sulfate binding site) are of 99%, 92% and 99%, respectively. Except for Val111, these values are similar to those observed in the multi-alignment of the dioxygenases superfamily (469 proteins). However, we have detected the consensus sequence D_68_-N_29_-L_100_-W_87_-A_98_-V_54_-H_100_-T_58_-N_99_-x(0,1)-A_27_-Y_81_-x(0,2)-D_98_-Y_96_ (subscript numbers indicate the percentage of conservation in the alkylsulfodioxygenase alignment; S3 Fig). This consensus corresponds to the residues Asp156 to Tyr168 in the reference sequence Q9WWU5. From this consensus sequence, we have defined the PROSITE-like pattern [DEN]-[NQTSKRGAED]-L-[WRV]-[AV]-[VLIMRTE]-H-[TSGDN]-[NF]-x(0,1)-{SGNFWYCMI}-[YFAG]-x(0,2)-[DES]-[YLQH]. This pattern has recovered 668 sequences from the TREMBL databank (July 2016), all annotated as "Dioxygenase", "Alkylsulfatase" or "Uncharacterized protein" (including the 111 sequences contained in the AtsK clade of the phylogenetic tree). A logo sequence was built using the multi-alignment of alkylsulfatases (Fig 6A).

Contrary to the FGy-sulfatases that are found throughout the tree of life (with the exception of land plants), the alkylsulfodioxygenases have been found only into three bacterial phyla. Of the 111 alkylsulfodioxygenases detected by phylogenetic analysis, 58 belong to the phylum *Proteobacteria*, 50 belong to the phylum *Actinobacteria* and three sequences to the phylum *Cyanobacteria*. Among the *Proteobacteria*, the class *betaproteobacteria* is represented by 28 sequences all belonging to the order *Burkholderiales*. There are 17 sequences from *Gammaproteobacteria* that all belong to the order *Pseudomonadales*. The class *Alphaproteobacteria* is represented by 12 sequences that belong essentially to the order *Rhizobiales*. Finally, one sequence is a *Deltaproteobacteria* (*Myxococcales*). Concerning the phylum *Actinobacteria*, all sequences come from the class *Actinobacteria* where 64% of sequences belong to the order *Corynebacteriales*. The other sequences from the class *Actinobacteria* are divided among the orders *Streptosporangiales* (6 sequences), *Streptomycetales* (5 sequences), *Micrococcales* (4 sequences), *Pseudonocardiales* (3 sequences) and *Catenulisporales* (2 sequences). The taxonomic positions of *Actinobacteria* and *Proteobacteria* indicate that the alkylsulfodioxygenases derived from fresh water or soil bacteria. No alkylsulfodioxygenases originated from eukaryotic organisms nor from marine prokaryotic organisms.

### Analyses of alignments from sulfatases belonging to the zinc-dependent beta-lactamase superfamily and phylogenetic analysis (families S3 and S4)

**Fig 7:**
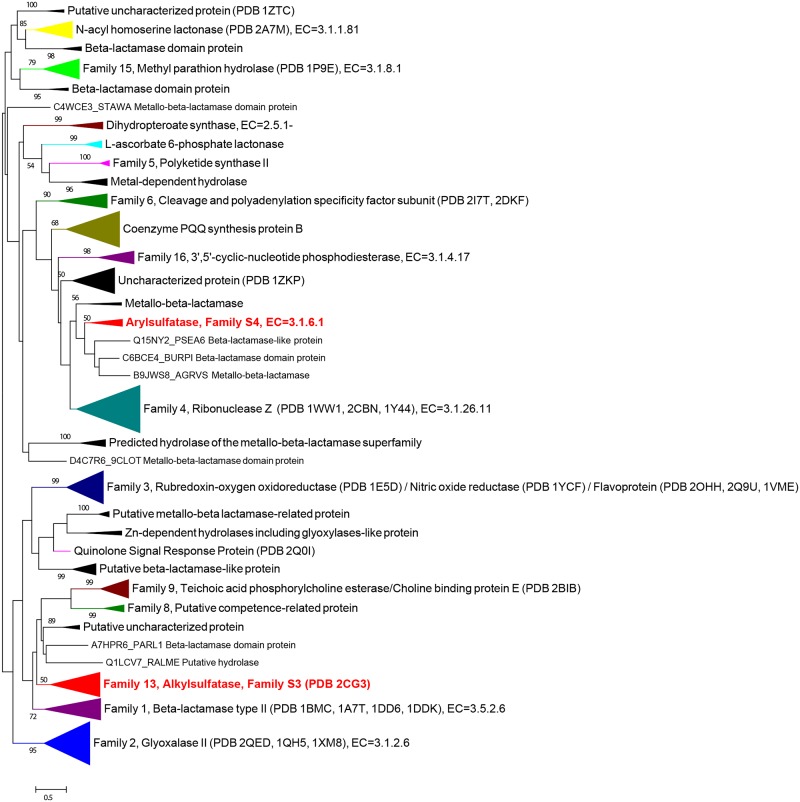
Phylogenetic tree of zinc metallo-β-lactamase superfamily. The tree was obtained by maximum likelihood with RAxML using the substitution matrix WAG from 96 positions of an alignment of 288 sequences belonging to various families of the superfamily of zinc metallo-beta-lactamase. The coloured clades contain the sequences with known activities. The black clades and the isolated sequences (not supported by high bootstrap values) contain no biochemically-characterized enzymes. The families S3 and S4 of sulfatases are shown in red. All the resolved three-dimensional structures are indicated. The numbers of the family are references to Daiyasu's groups [79]. Only bootstrap values above 50% are shown.

The desulfation of alkyl-compounds is not restricted to the alkylsulfodioxygenases. The first alkylsulfohydrolase, SdsA1, was characterized from *Pseudomonas aeruginosa* PAO1 [26]. SdsA1 belongs to the zinc metallo-β-lactamase superfamily. On the basis of sequence similarities and biological functions, this superfamily was divided in 16 families [79]. All members of this superfamily are characterized by the same fold and by the catalytic signature HxHxDH where the aspartate and histidine residues are involved in cationic metal coordination (Fig 1F). A multi-alignment was obtained from a sample of 288 sequences belonging to various families within the zinc metallo-β-lactamase superfamily. Due to high sequence divergence, the phylogenetic trees were built from only 96 positions from this alignment. Nonetheless this multiple alignment included the five conserved segments previously described by Daiyasu and coworkers [79]. The alkylsulfohydrolase family, which in this sample included 17 sequences, was easily identified (Fig 7). A BLASTP search using SdsA1 and the 17 alkylsulfohydrolase sequences as query sequences recovered 370 putative sulfatases in the UniProt databank. From the three-dimensional structure of the SdsA1 alkylsulfohydrolase (PDB 2CFU), Hagelueken and coworkers have identified that the sequence A_80_-**E**_84_-N_57_-A_37_-T_51_-H_53_-T_48_-L_51_-H_84_-**N**_98_-L_42_-Y_61_-**T**_81_-L_73_-**R**_99_-G_99_-A_83_-K_41_-V_54_-**R**_99_ forms the loop responsible for sulfate binding [65] (from Ala298 to Arg317 in the reference sequence SdsA1 Q9I5I9; subscript numbers indicate the percentage of conservation in alignment; amino acids involved in the binding sulfate are in bold; S4 Fig). On the basis of this initial consensus sequence and excluding the sequences responsible for small insertions present in this loop (15 sequences), we have defined this updated consensus pattern [AGSIT]-[EDNA]-[NDLVTECISM]-x(4)-[LMFQIWVY]-[HP]-[NDQA]-[LIFVTP]-x-[TASPD]-[LIPFMV]-[RCT]-G-[ATDSGLVE]-x(2)-R. This pattern recovered about 2000 sequences from the TREMBL databank (July 2016), mostly annotated as "Alkyl sulfatase or beta-lactamase", "Metallo-beta-lactamase superfamily protein" or "Uncharacterized protein". This collection contained 95% of sequences present in our alignment. Only 7 false positive sequences were identified among all recovered sequences. A logo sequence was built using the multi-alignment of alkylsulfohydrolases (Fig 6B).

The alkylsulfohydrolases are ubiquitous enzymes and are present in the three kingdoms of life. Among the 370 alkylsulfatases detected, three sequences derived from *Archaea* belonging to the phylum *Euryarchaeota* (represented by one halophilic strain and two methanogenic strains) and 31 from Eukaryota (3 Alveolata, 10 Amoebozoa, 17 fungi ascomycetes and only one Metazoa [*Tricoplax adhaerens*]). The other sequences belong to the kingdom *Bacteria*. Seventy-six sequences originate from Gram-positive strains of which 52 *Actinobacteria* (belonging overwhelmingly to the order *Corynebacteriales*) and 24 *Firmicutes*, twelve belonging to the class *Clostridia*, nine to the class *Bacilii* and three to the class *Erysipelotrichi*. The Gram-negative bacteria provided 259 sulfatase sequences. With the exception of one *Acidobacteria*, one *Cyanobacteria* (order *Chroobacteria*), two *Bacteroidetes* (order *Bacteroidia*), four *Fusobacteria* (family *Leptotrichiaceae*) and six *Planctomycetes*, the other sequences all belong to the phylum *Proteobacteria*. Within this later phylum, 33 sequences belong to the class *Alphaproteobacteria*, 19 *Betaproteobacteria* (all from order *Burkholderiales*) and 5 to the class *Deltaproteobacteria*. The remaining 188 sequences belong to the class *Gammaproteobacteria*, represented essentially by the families *Enterobacteriaceae*, *Vibrionaceae*, *Shewanellaceae* and *Pseudomonadaceae*. However, the number of species in these families is low. The family *Enterobacteriaceae* is essentially represented by various strains of *Escherichia coli* and by different subspecies of *Salmonella enterica*. The family *Vibrionaceae* is mainly represented by various strains of *Vibrio cholerae*. In contrast, the families *Shewanellaceae* and *Pseudomonadaceae* are represented by many species from the genera *Shewanella* and *Pseudomonas* respectively. Finally, 13 sequences of putative alkylsulfohydrolases originated from unidentified *Gammaproteobacteria* and only one sequence is present in the *Paramecium bursaria Chlorella* virus FR483. The alkylsulfohydrolases are mainly produced by saprophytic organisms from soil or fresh water or by pathogenic organisms. In contrast to the alkylsulfodioxygenases, alkylsulfohydrolases are nonetheless present in the marine environment as deduced by the sequences belonging to the phylum *Planctomycetes* or by the high representation of the order *Alteromonadales* (families *Shewanellaceae*, *Moritellaceae*, *Colwelliaceae* and *Psychromonadaceae*).

Due to its high capacity to hydrolyze the 4-methylumbelliferyl sulfate (4MUF-S), the protein AtsA from *Pseudoalteromonas carrageenovora* 9^T^ was described as an arylsulfohydrolase [27]. This protein displays the catalytic HxHxDH motive indicating it belongs to the zinc metallo-β-lactamase superfamily, as previously suggested by Melino and coworkers [66]. Except for the catalytic residues, AtsA possesses very limited sequence identity with the alkylsulfohydrolases (~13%). However, AtsA shows about 30% similarity with the members of the ElaC family (ribonuclease Z family) within the zinc metallo-β-lactamase superfamily. This observation was confirmed by our phylogenetic analysis of the zinc metallo-β-lactamase superfamily in which AtsA and four related proteins constitute a clade close to the ribonuclease Z clade (Fig 7). The other putative arylsulfohydrolases present in the UniProt databank were identified by BLAST search, using AtsA as query sequence. Only fifteen sequences could be new putative arylsulfohydrolases. To verify their position, these 15 sequences were aligned with 225 sequences belonging to the ElaC/AtsA family. A maximum likelihood phylogenetic tree was built from 187 aligned positions. The resulting tree shows that the 15 putative arylsulfatases form a clade that remains close to that of RNase Z (S5 Fig). The organisms that encode for this putative activity are all *Bacteria* belonging to the phylum *Proteobacteria*. The class *Alphaproteobacteria* is the most represented with the genera *Novosphingobium*, *Sphingobium* and *Maritimibacter*. Some *Betaproteobacteria* are also found (genus *Ralstonia* and *Comamonas*). Finally, *Gammaproteobacteria* are represented by the genus *Pseudoalteromonas*. The genera *Pseudoalteromonas* and *Maritimibacter* seem to be the only representatives from the marine environment. It is interesting to note that the species belonging to the genera *Sphingobium* and *Novosphingobium* are commonly isolated from soil and they can degrade a variety of chemical compounds such as aromatic, chloroaromatic and phenolic compounds.

## Discussion

### Proposition of nomenclature and classification for sulfatases

With the increasing number of completely sequenced genomes, new sulfatase genes and their corresponding proteins have been regularly released into sequence databases, but their functional annotation is often prone to inaccuracies and misinterpretations due to several reasons. The formylglycine-dependent sulfatases are frequently considered as the only family of sulfatases, even in recent articles or reviews, and are thus annotated as “sulfatases” or “arylsulfatases” without any other precisions. This error is erroneously propagated by two popular web sites, PROSITE and PFAM, which provide protein profiles reducing the sulfatases to FGly-sulfatases (http://www.expasy.ch/prosite/PDOC00117 and http://pfam.xfam.org/family/PF00884, respectively). These signatures also correspond to the profiles IPR000917 (http://www.ebi.ac.uk/interpro/entry/IPR000917) and IPR024607 (http://www.ebi.ac.uk/interpro/entry/IPR024607) in the Interpro database [80]. More surprisingly, the “seed” on which is based the PFAM profile PF00884 comprises numerous uncharacterized sequences which do not feature the catalytic signature of FGly-sulfatases! For instance, eleven sequences homologous to a putative protein from *Streptococcus mutans* (trEMBL accession: Q840W2) contain a conserved TXNXE motif instead of the canonical (C/S)xPxR pattern. Among the 59 sequences composing the PFAM seed, 30 putative proteins featured a threonine in place of the catalytic cysteine or serine. To the best of our knowledge, oxidation of a threonine residue, in a similar manner to serine or cysteine, would give the corresponding ketone, not formylglycine residue. It is probably the reason why it has never been shown that the formylglycine residue can be generated from a threonine. Nonetheless none of the TXNXE-containing proteins have been characterized yet, and they cannot be considered as functional sulfatases in absence of experimental evidences. Therefore, the profile PF00884 is incorrect and has already introduced numerous false annotations in sequence databases. Another problem is the inaccurate use of the term “arylsulfatase”. Artificial aryl compounds such as 4MUF-S, p-nitrophenyl-sulfate (PNP-S) and p-nitrocatechol sulfate (PNC-S) are conveniently used to test the activity of new sulfatases, but are not the true substrates of these enzymes. For instance, the so-called “arylsulfatases” ARSA and ARSB are specific for cerebroside-sulfate and N-acetylgalactosamine-4-sulfate, respectively, which are not phenolic compounds (Table 1). Finally, the number of sulfatases with known substrate specificity is limited in comparison to the huge diversity of sulfated compounds. Moreover, most of these enzymes were characterized in animals and only in a few bacterial phyla. Since genome annotations are generally based on best BlastP hits against sequence databases, new sulfatases are often given substrate specificities which are not always relevant for non-model organisms. The presence of such inexact annotations in databases creates a snowball effect propagating assignment errors [81]. A classification system reflecting the catalytic machinery, allowing for a better prediction of substrate specificity and for setting the limit of functional annotations, is therefore urgently needed for sulfatases.

We propose to classify the sulfatases according to the principles used for the classification of carbohydrate-active enzymes (http://www.cazy.org/) [82] and of peptidases (http://merops.sanger.ac.uk/) [83]. Each sulfatase is assigned to a **Family** on the basis of a significant similarity in amino acid sequence. Sulfatases belonging to the same family derive from a common ancestor, adopt a similar fold and display conserved catalytic residues. Because the fold of proteins is better conserved than their primary structure, some families of sulfatases can be grouped in **Clans** if they share a common fold and catalytic machinery [84]. Based on these principles, four families of sulfatases can be currently defined. Due to their abundance and biological importance we naturally define the formylglycine-dependent sulfatases as the family 1 of sulfatases, referred to as family S1. To respect the order suggested by Hagelueken and coworkers [65], we propose to formally define the families 2 (family S2), 3 (family S3) and 4 (family S4) as comprising the homologues of the alkylsufatase (alkylsulfodioxygenase) AtsK from *P*. *putida* S-313 [23, 25], of the alkylsulfatase (alkylsulfohydrolase) SdsA1 from *P*. *aeruginosa* PAO1 [26, 65] and of the arylsulfatase (arylsulfohydrolase) AtsA from *P*. *carrageenovora* 9^T^ [27], respectively. Moreover, the alkylsulfatase SdsA1 and the arylsulfatase AtsA both belong to the zinc metallo-beta-lactamase superfamily and feature conserved catalytic residues despite their weak sequence identity (Fig 7 and S5 Fig) [65, 66]. Consequently, we propose to group families S3 and S4 into Clan S_A of sulfatases. Families S2, S3 and S4 of sulfatases each comprise only one characterized sulfatase and are found by default to be monospecific (containing only one EC number). In contrast, Family S1 is highly polyspecific, currently with ten official EC numbers (Table 1). Simple membership to this family is thus not sufficient to correctly forecast the exact specificity of new FGly-sulfatases. The definition of **Subfamilies** allowing a better prediction of substrate specificity is also needed and will be detailed in the following paragraph.

### Classification of Family S1 formylglycine-dependent sulfatases into substrate-specific subfamilies

The survey of FGly-sulfatases in genomic data indicates that these genes are frequent in bacteria and eukaryotes, but usually present in a few copies per species, which indicates a moderate functional diversification. Large multigenic families of FGly-sulfatases are only observed in some marine heterotrophic bacteria, and to a lesser extent in vertebrate gut bacteria. Sulfur scavenging is less essential for marine microbes than for freshwater and terrestrial microorganisms, given that seawater is rich in inorganic sulfate (~28 mM) [55]. On the other hand, the marine environment offers an unmatched diversity of sulfated biomolecules. Some compounds are common to the terrestrial environment, such as GAGs from fishes and marine invertebrates and mammals, but other sulfated molecules are unique to marine organisms, especially in marine algae and seagrasses. For instance, the numerous FGly-sulfatases of *R*. *baltica* and *Z*. *galactanivorans* are likely involved in the utilization of these various sulfated compounds as carbon sources. *Z*. *galactanivorans* Dsij^T^ is already known for its capacity to degrade agars [85, 86], porphyrans [87] and carrageenans [88, 89]. Moreover, we have demonstrated that *R*. *baltica* SH1^T^ also degrades κ- and ι-carrageenans [90]. These marine proteins likely cover an unprecedented panel of substrate specificities and constitute a significant fraction of FGly-sulfatases in sequence databases. The correct annotation of these enzymes is thus essential to avoid error propagation in sequence databases and to define substrate specific subfamilies of FGly-sulfatases.

The phylogenetic tree of the FGly-sulfatases is divided into 73 different clades (S2 Fig). The bootstrap analyses and the different tests performed confirmed the solidity of these clades. Interestingly, the 36 sequences with known substrate specificity, which mainly originate from mammals, do not follow the taxonomy but mainly cluster in accordance to their substrate specificity (S2 Fig). This tendency is clear for the genuine arylsulfatases (clade 4), the N-sulfoglucosamine sulfohydrolase (SGSH, clade 8), the iduronate 2-sulfatase (IDS, clade 7), the mucin-desulfating sulfatase (MdsA, clade 11), the N-acetylglucosamine 6-sulfatase GNS and the sulfatases SULF1 and SULF2 (clade 6). Interestingly, the sulfatases MdsA, GNS, SULF1 and SULF2, which form the two sister clades 6 and 11, are all specific for N-acetylglucosamine-6-sulfate but in different biological contexts: (i) the lysosomal sulfatase GNS is an exo-hydrolase required for the degradation of heparan-sulfate and keratan-sulfate [41]; (ii) SULF1 and SULF2 are extracellular endo-sulfatases regulating Wnt signalling through desulfation of cell surface heparan sulfate proteoglycans [44, 45]; (iii) the bacterial sulfatase MdsA is involved in the catabolism of host mucin glycoproteins [51]. Based on the high bootstrap values observed for the deep nodes in the neighborhood of clades 6 and 11, it is probable that the small clades 25, 34, 35 and 36 are also specific for the N-acetylglucosamine-6-sulfate, in unknown contexts. Conversely, the sulfatases GNS, IDS and SGSH, which act on different sugar monomers of heparan sulfate, emerge into distinct clades (S2 Fig). Similarly, the chondroitin sulfatases ARSB and GALNS do not group together (clades 2 and 5 respectively), likely due to their difference in regioselectivity (N-acetylgalactosamine 4-sulfate and 6-sulfate, respectively). Therefore, the promiscuity between carbohydrate sulfatases is more dictated by the type of sugar monomer and by the sulfate position than by the overall nature of the polysaccharide. More surprisingly, the iduronate 2-sulfatases from *M*. *musculus* and *Pedobacter heparinus* do not cluster together (clades 7 and 9 respectively), whereas they display similar substrate specificity (S2 Fig). A closer look reveals that these proteins share only 22% of sequence identity, suggesting that this activity independently emerged several times during the divergence of FGly-sulfatases. Such convergent evolution within the speciation of a protein family has been already observed for xylan-specific CBM6s [91].

Nevertheless, the phylogenetic position of some FGly-sulfatases apparently contradicts this tendency to cluster according to enzymatic activities; for example, clade 2 (N-acetylgalactosamine-4-sulfatases) groups with clade 10 (composed of three alleles of glucosinolate sulfatase from *Plutella xylostella*) whereas they catalyze different reactions (S2 Fig, S1 File). It is noteworthy that the closest homologues of the glucosinolate sulfatases group unexpectedly with ARSB. The glucosinolate sulfatase is an orphan sequence, suggesting that this gene is unique to the Diamondback moth and emerged by duplication of an ancestral ARSB gene. A second similar situation exists with clades 7 (iduronate 2-sulfatases) and 66 (S2 Fig). However, since the substrate specificity of this latter clade is unknown it is possible that these sequences, although showing only 26% of sequence identity with the IDS sequence, also harbor an iduronate 2-sulfatase activity or a closely related activity. There remain the two cases of clades 14 and 19, each clade supported by low bootstrap values (S2 Fig). As mentioned in the results section, it is possible that these clades correspond to multiple substrate specificities. For example, the sequence Q15XH3 from *P*. *atlantica* T6c, which is localized in clade 19, has been recently described as an endo-4S-iota-carrageenan sulfatase that converts iota-carrageenan into alpha-carrageenan by desulfation of the C4 sulfated D-galactose moiety [92]. Within this clade, this enzyme forms a sub-clade (bootstrap value 100%) with the sequences G0L000, F0RBY4 and E6XAT3 from the marine flavobacteria *Z*. *galactanivorans* Dsij^T^, *Cellulophaga lytica* DSM 7489^T^ and *C*. *algicola* IC166^T^, respectively (S1 File). Similarly, it has also been recently described that the protein Q15XG7 from *P*. *atlantica* T6c is a endo-4S-kappa-carrageenan sulfatase that removes the C4 sulfate from the D-galactose of kappa-carrageenan, converting this substrate to beta-carrageenan [93]. Within clade 19, Q15XG7 also forms a sub-clade (bootstrap value 100%) which includes sequences E6X9N5, E6XA77, F0RIB9, F0RBY9 and G0L4M9 from the same bacteria that form the Q15XH3 sub-clade within clade 19 (S1 File). All these enzymes likely desulfate the D-galactose-4-sulfate from carrageenan. But this hypothesis is probably not true for the entire clade 19. Indeed, this clade contains not only marine bacteria but also some terrestrial or freshwater bacteria including *Chthoniobacter flavus*, *Flavobacterium johnsoniae* or *Sphingobacterium spiritivorum* which are unlikely to desulfate carrageenan.

Altogether, the general clustering of the characterized FGly-sulfatases seems to indicate that the clades observed in the phylogenetic tree correspond to subfamilies representing different substrate specificities. Such polyspecificity within a family has been demonstrated for other protein classes, for instance for glycoside hydrolases (e.g. families GH16 [88], GH13 [94], GH5 [95]) and for carbohydrate binding modules (e.g. CBM6 [91], CBM32 [96, 97]). Thus, we can confidently predict that the sequences that group with characterized FGly-sulfatases have similar substrate specificities. However, we have also unraveled sixty clades which do not possess any characterized FGly-sulfatases. The principles underlying the clustering of the known FGly-sulfatases are logically valid for these additional clades. Therefore, our analysis supports the existence of at least 60 subfamilies of FGly-sulfatases with novel, unidentified substrate specificities.

**Fig 8:**
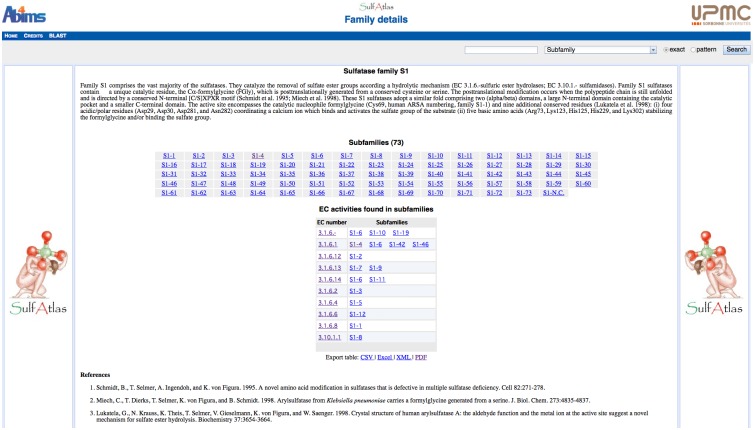
SulfAtlas website. Example of the “sulfatase family S1” page. Within each family, a text presents the current knowledges of concerned sulfatases and for the S1 family, the list of subfamily is shown and also the distribution of known EC numbers within them.

To summarize, we recommend abandoning the systematic use of the misleading term “arylsulfatase” and to restrict it to enzymes truly specific for natural phenolic compounds (EC 3.1.6.1), such as steroid-sulfate [2], sulfated flavonoids [6] or lignin-derived sulfated phenols [55]. For the annotation of new sulfatases, we suggest using the generic term “sulfatase”, followed by the mention of the family (e.g. sulfatase, Family S3). For the family S1 (FGly-sulfatases), we propose defining substrate-specific subfamilies on the basis of our present phylogenetic analysis (S2 Fig). A subfamily will be referred with an additional digit after the number designing the family using an underscore as separation (i.e. Family S1_n). We have attributed the first numbers to the subfamilies comprising the currently characterized FGly-sulfatases, from S1_1 (cerebroside sulfatase, EC 3.1.6.8) to S1_12 (choline sulfatase, EC 3.1.6.6). The remaining subfamilies, from S1_13 to S1_72, correspond to clades of unknown substrate specificity. For the annotation of new FGly-sulfatases, we propose using either the known specificity when possible (for the subfamilies S1_1 to S1_12) or the generic term “sulfatase” (for the subfamilies S1_13 to S1_72), followed by the subfamily number: e.g. mucin-desulfating sulfatase, family S1_11 or sulfatase, family S1_23. The sequences included in the subfamily S1_0 possess the catalytic signature of the FGly-sulfatases and also belong to the superfamily of alkaline phosphatases. They have been shown to indeed display a FGly, but in reality they are phosphonate monoester hydrolases/phosphodiesterases (EC 3.1.-.-) [98]. Their significant level of sequence similarities with the FGly-sulfatases and the presence of a catalytic FGly suggest that these two enzyme classes share a common ancestor. The S1_0 sequences were thus used as outgroup in our phylogenetic analysis. When new subfamilies will be discovered, they will be added to this classification and sequentially numbered. Moreover, the clades with unknown specificity have been defined on a rather conservative basis (deepest node with a reliable bootstrap value), resulting in rather large subfamilies. If one day two FGly-sulfatases from the same subfamily are experimentally demonstrated to have different activities, the subfamily will be split on the basis of the deepest reliable node resulting into two monospecific subfamilies. To avoid instability in the classification, the subfamily with the first demonstrated activity will keep the number of the original subfamily, while the second subfamily will be given a new, sequential number. To provide this classification system to the scientific community, we have built a free web accessible database, called SulfAtlas, available at the following address: http://abims.sb-roscoff.fr/sulfatlas/. The home page of the SulfAtlas website summarizes information about the different families of sulfatases, giving the number of sulfatases in each of them. Clicking on a family name (e.g S1) displays the family page with information about the family, the list of its subfamilies and the list of EC numbers found in these subfamilies (Fig 8). The subfamily page, accessed by clicking on a subfamily name, shows some subfamily descriptors (known enzymatic activities, catalytic residues and available 3D structures) and a table with all the UniProt accession numbers of sulfatases belonging to this subfamily with, for each enzyme, the protein or locus name, the EC number, the taxonomic name of organism and the PDB accession number when it exists. All these fields are linked to the matching databases: UniProtKB from UniProt, the enzyme database ExplorEnz, the Taxonomy database from NCBI and the Protein Data Bank from RCSB PDB. Selected sulfatase sequences can also be exported in fasta format. Moreover, it is possible to search the database using keywords: the family or subfamily number, the taxonomy ID number, the organism name, the locus or gene name, the full or short UniProt accession number (ex. G0L000_ZOBGA or G0L000 respectively) or the EC number and the PDB accession number. Finally, it is possible to query SulfAtlas by single BLAST or multiple BLAST with one sequence or with an entire proteome. Updating of SulfAtlas will be facilitated by the use of different consensus patterns (used alone or in combination) identified in multiple alignments (Figs 4 and 6).

### Evolution of sulfatases

The existence of four sulfatase families suggests that this activity independently appeared at least four times during the evolution of life. It is reasonable to think that sulfatase activity comes from duplication of ancestral genes. This assumption derives from fact that sulfatase activity is present in Fe(II) alpha-ketoglutarate-dependent dioxygenase, zinc-dependent beta-lactamase and alkaline phosphatase superfamilies, where the members within each superfamily have in common either fold, catalytic amino acids or reaction mechanism. The sulfatase families S2 and S3 are derived from the Fe(II) alpha-ketoglutarate-dependent dioxygenase and zinc-dependent beta-lactamase superfamilies, respectively. The only activity known for both families is alkylsulfatase activity. The most likely role for these enzymes is in the absorption of sulfate ions using detergents as a sulfur source, present in water or soil contaminated by effluent from car wash waste water, laundry detergent or shampoo. The sulfatase family S2 is only composed of bacteria that live in fresh water or soil belonging in equal parts to the classes *Actinobacteria* (Gram positive) and *Alphaproteobacteria* (Gram negative). The family S3 sulfatases are present in the three kingdoms of life, although the archaeal and eukaryotic representatives are very rare. More than 90% of the family S3 sequences belong to bacteria from the class *Gammaproteobacteria*, in families *Enterobacteriaceae* or *Vibrionaceae*. These bacterial families are not represented among bacteria possessing family S2 sulfatases. The bacteria with family S3 sulfatases are likely opportunistic microbes desulfating phenolic compounds naturally occurring in terrestrial and marine environments, while those with the family S2 sulfatases might be considered as true bacterial “cleansers” of soil.

Finally, the family S4 is represented by a very small number of members. Only arylsulfatase activity has been detected using an artificial substrate. Thus, it is difficult to predict the actual function *in vivo*. However, it is possible to postulate that these enzymes have arisen from a gene duplication of a gene belonging to the family elaC and might play a role in the uptake of sulfate from phenolic compounds present in soil (by the *Alphaproteobacteria*) or marine sediments (by some marine *Gammaproteobacteria*).

Formyglycine-dependent sulfatases share a common structural framework and catalytic machinery, but display an exceptional diversity of substrate specificity. The functional diversification of FGly-sulfatases is mainly due to gene duplication, the new-born paralogs escaping the pressure of pre-existing constraints and becoming free to evolve new specificities [99]. Most of these gene duplications likely occur early in both bacterial and eukaryotic evolution, as shown by the high sequence divergence between the various types of FGly-sulfatases (Table 2). Our phylogenetic analyses indicate that these proteins have diverged from a common ancestor into clades reflecting their substrate specificity. The apparent incongruence between the phylogenetic tree of FGly-sulfatases and species tree is mainly explained by the polyspecificity of this protein family and the high sequence divergence between FGly-sulfatases of different substrate specificities (Table 2). Thus it is difficult to establish a general scenario for the evolution of FGly-sulfatases by only phylogenetic approaches. Nonetheless, the distribution of these enzymes in the tree of life gives some evolutionary hints. FGly-dependent sulfatases are widespread in bacteria and eukaryotes (S2 Fig), whereas they are only found in two archaeal classes, *Methanomicrobia* and *Halobacteria*, both belonging to the *Euryarchaeota* phylum, which encompasses mesophilic methanogenic or halophilic archaea. It is noteworthy that phylogenomics data supports a hyperthermophilic and non-methanogenic ancestor to extant archeal lineages and that mesophily is a secondary adaptation for *Archaea* [100]. The paucity and the distribution of FGly-sulfatases in *Archaea* suggest that these microorganisms acquired FGly-sulfatases through horizontal gene transfer (HGT) from mesophilic bacteria. Consequently the archaeal/eukaryotic common ancestor likely lacked FGly-sulfatases, assuming *Archaea* and Eukaryota are sister groups, as is widely held [100, 101]. The most parsimonious scenario is that FGly-sulfatases have a bacterial origin and were transmitted to eukaryotes by endosymbiotic gene transfer (EGT) from the alpha-proteobacterial progenitor of the mitochondria [102]. Therefore, the absence of FGly-sulfatases in some eukaryotic phyla is best explained by gene loss after the mitochondrial endosymbiosis.

## Supporting Information

S1 Fig Identified consensus sequences in the global multi-alignment of FGly-sulfatases. The global multi-alignment was composed of 4058 FGly-sulfatases aligned with MAFFT program using the L-INS-i algorithm as iterative refinement method. The consensus sequences (in bold) corresponding to the catalytic site (PROSITE signature PS00523), the PROSITE signature PS00149, the two calcium binding sites and to a supplementary signature, are shown in A and B C D and E respectively. Amino acids involved in calcium binding and catalytic amino acids are shown in red in consensus sequences. The blue numbers indicate the position of amino acids in the reference sequence AtsA (P51691). For each position, the present amino acids and the percentage of sequence that they represent in multi-alignment are indicated. The value 0% means that the amino acid is present in less than 1% of sequences. The accession numbers of sequences responsible of insertions in the consensus sequence or their number is indicated at positions "x".
(PDF)

S2 Fig Phylogenetic tree of FGly-sulfatases. The tree was obtained by maximum likelihood with RaxML using MTMAMF as a substitution matrix from an alignment of 4005 sequences and 329 positions. The clades represent the subfamilies according the proposed nomenclature. For the subfamilies with a characterized activity, the activity name and the corresponding EC number are indicated. All resolved three-dimensional structures are indicated. Orphean sequences (which were not include in a clade) are annotated S1_N.C. (for non-classified). Only bootstrap values above 60% are shown.
(PDF)

S3 Fig Consensus sequences extracted from the global multi-alignment of Alkylsulfodioxygenases. The alkylsulfodioxygenases consensus sequence was deduced from an alignment of 111 sequences, extracted from the global multi-alignment of 469 dioxygenases. This latter alignment was obtained using the MAFFT program with the L-INS-i algorithm as the iterative refinement method. The consensus sequence appears in bold. The blue numbers indicate the position of amino acids in the reference sequence AtsK (Q9WWU5). For each position, the amino acids present and the percentage of sequence that they represent in the multi-alignment are indicated. The value 0% means that the amino acid is present in less than 1% of sequences. The accession numbers of sequences responsible of insertions in the consensus sequence or their number is indicated at positions "x".
(PDF)

S4 Fig Consensus sequences extracted from the global multi-alignment of Alkylsulfohydrolases. The alkylsulfohydrolases consensus sequence was deduced from an alignment of 370 sequences obtained using the MAFFT program with the L-INS-i algorithm as iterative refinement method. The consensus sequence appears in bold. The blue numbers indicate the position of amino acids in the reference sequence SdsA1 (Q9I5I9). Amino acids involved in binding sulfate are shown in red in the consensus sequences. For each position, the amino acids present and the percentage of sequence that they represent in the multi-alignment are indicated. The value 0% means that the amino acid is present in less than 1% of sequences.
(PDF)

S5 Fig Phylogenetic tree of the ElaC/AtsA family. The tree was obtained by maximum likelihood with RAxML using the substitution matrix WAG from 187 positions from an alignment of 240 sequences. The blue clade contains the characterized tRNases Z (EC 3.1.26.11) and related sequences. The black clades and the isolated sequences (not supported by high bootstrap values) contain no biochemically-characterized enzymes. The family S4 of the sulfatases is shown in red. All the resolved three-dimensional structures are indicated. Only bootstrap values above 50% are shown. The sequence belonging to the S3 family of sulfatases Q9I5I9 (SdsA1 from *Pseudomonas aeruginosa* PAO1) was used as an outgroup.
(PDF)

S1 File MEGA 5 source file (.mts) corresponding to the non-collapsed phylogenetic tree of FGly-sulfatases (family S1, 4058 sequences) as shown in S2 Fig. (MTS)
