# Supplementary material for: Matching the Diversity of Sulfated Biomolecules: Creation of a Classification Database for Sulfatases Reflecting Their Substrate Specificity
Source: PLoS One. 2016 Oct 17;11(10):e0164846. doi: 10.1371/journal.pone.0164846 (PMC5066984; doi:10.1371/journal.pone.0164846)
Supplement: S1 Fig — The global multi-alignment was composed of 4058 FGly-sulfatases aligned with MAFFT program using the L-INS-i algorithm as iterative refinement method. The consensus sequences (in bold) corresponding to the catalytic site (PROSITE signature PS00523), the PROSITE signature PS00149, the two calcium binding sites and to a supplementary signature, are shown in A and B C D and E respectively. Amino acids involved in calcium binding and catalytic amino acids are shown in red in consensus sequences. The blue numbers indicate the position of amino acids in the reference sequence AtsA (P51691). For each position, the present amino acids and the percentage of sequence that they represent in multi-alignment are indicated. The value 0% means that the amino acid is present in less than 1% of sequences. The accession numbers of sequences responsible of insertions in the consensus sequence or their number is indicated at positions "x". (PDF) [file pone.0164846.s001.pdf]

# (A)

## Catalytic site (PROSITE signature PS00523)

| 49 | | 50 | | 51 | | 52 | | 53 | | 54 | | 55 | |
|---|---|---|---|---|---|---|---|---|---|---|---|---|---|
| | % | | % | | % | | % | | % | | % | | % |
| **P** | **43** | **V** | 26 | **C** | **79** | **S** | **29** | **P** | **87** | **S** | **55** | **R** | **99** |
| A | 22 | L | 23 | S | 21 | T | 24 | A | 9 | T | 20 | L | 0 |
| S | 15 | I | 11 | | | A | 16 | S | 0 | A | 13 | I | 0 |
| G | 4 | S | 10 | | | G | 11 | T | 0 | G | 6 | Q | 0 |
| Q | 4 | T | 8 | | | C | 7 | V | 0 | F | 1 | K | 0 |
| T | 3 | M | 5 | | | M | 2 | I | 0 | H | 0 | | |
| N | 2 | P | 4 | | | V | 2 | Q | 0 | C | 0 | | |
| M | 1 | A | 3 | | | H | 0 | G | 0 | N | 0 | | |
| V | 1 | R | 2 | | | I | 0 | C | 0 | Y | 0 | | |
| L | 0 | Q | 0 | | | N | 0 | M | 0 | I | 0 | | |
| C | 0 | N | 0 | | | Q | 0 | N | 0 | V | 0 | | |
| R | 0 | K | 0 | | | L | 0 | E | 0 | M | 0 | | |
| H | 0 | Y | 0 | | | W | 0 | | | Q | 0 | | |
| Y | 0 | F | 0 | | | R | 0 | | | W | 0 | | |
| E | 0 | G | 0 | | | E | 0 | | | | | | |
| I | 0 | E | 0 | | | K | 0 | | | | | | |
| | | W | 0 | | | Y | 0 | | | | | | |
| | | D | 0 | | | F | 0 | | | | | | |
| | | H | 0 | | | | | | | | | | |

| 56 | | 57 | | | 58 | | 59 | | 60 | | 61 | |
|---|---|---|---|---|---|---|---|---|---|---|---|---|
| | % | | % | | | % | | 75 | | % | | % |
| **A** | 53 | **S** | **33** | **x(0-6)** | **L** | **56** | **L** | **46** | **T** | **83** | **G** | **98** |
| S | 15 | A | 2 | A9UYU7 | I | 17 | M | 21 | S | 11 | S | 0 |
| Y | 4 | G | 8 | | F | 13 | I | 11 | A | 1 | A | 0 |
| G | 4 | M | 8 | | M | 8 | F | 6 | L | 1 | C | 0 |
| T | 3 | T | 7 | | V | 2 | Y | 3 | M | 0 | | |
| F | 3 | C | 4 | | T | 0 | W | 2 | R | 0 | | |
| C | 3 | V | 2 | | Y | 0 | H | 2 | G | 0 | | |
| V | 3 | E | 2 | | A | 0 | V | 2 | C | 0 | | |
| R | 2 | Q | 2 | | C | 0 | Q | 1 | Y | 0 | | |
| M | 1 | N | 1 | | W | 0 | A | 0 | F | 0 | | |
| N | 1 | D | 0 | | S | 0 | T | 0 | N | 0 | | |
| Q | 1 | K | 0 | | Q | 0 | N | 0 | I | 0 | | |
| I | 0 | I | 0 | | H | 0 | K | 0 | H | 0 | | |
| P | 0 | L | 0 | | K | 0 | S | 0 | V | 0 | | |
| H | 0 | | | | | | C | 0 | E | 0 | | |
| L | 0 | | | | | | R | 0 | Q | 0 | | |
| K | 0 | | | | | | | | | | | |
| E | 0 | | | | | | | | | | | |
| W | 0 | | | | | | | | | | | |

**(B)**

**PROSITE signature PS00149**

| 105 | | | 106 | | 107 | | 108 | | | 109 | | | 110 | | | 111 | | | 112 | | | 113 | | | 114 | | | 115 | |
|---|---|---|---|---|---|---|---|---|---|---|---|---|---|---|---|---|---|---|---|---|---|---|---|---|---|---|---|---|---|
| | % | | | % | | | | % | | **x** | % | | **x** | % | | **x** | % | | | % | | | % | | | % | | | % |
| **G** | **94** | **x(0,1)** | **Y** | **93** | **x** | % | **T** | **84** | **x(0,42)** | **A** | **31** | **x(0,3)** | **L** | **18** | **x(0,13)** | **I** | **24** | **x(0,3)** | **G** | **95** | **x(0,5)** | **K** | **94** | **x(0,3)** | **W** | **59** | **x(0,2)** | **H** | **81** |
| D | 2 | Q8I1A5 | I | 2 | **A** | **12** | S | 3 | A7SK50 | G | 23 | A6C8W8 | M | 15 | A6C8W8 | F | 20 | (50 seq.) | S | 2 | (60 seq.) | D | 2 | (334 seq.) | M | 6 | (91 seq.) | L | 4 |
| N | 1 | B4NG05 | V | 1 | R | 12 | C | 3 | Q4SR77 | Y | 8 | A6DPF2 | A | 11 | A6DPF2 | V | 20 | | T | 0 | | R | 1 | | Y | 6 | | G | 3 |
| A | 0 | Q298E8 | W | 1 | Y | 11 | V | 2 | | H | 5 | D2R663 | Y | 10 | D2R663 | S | 8 | | A | 0 | | Y | 0 | | N | 6 | | N | 3 |
| S | 0 | B4G3P1 | F | 0 | H | 9 | N | 1 | | F | 4 | A6DPE8 | I | 7 | Q7UT91 | A | 8 | | N | 0 | | M | 0 | | L | 4 | | D | 1 |
| H | 0 | B4JGE7 | M | 0 | Q | 9 | A | 1 | | L | 3 | | Q | 6 | A6CEG6 | T | 4 | | Y | 0 | | L | 0 | | F | 3 | | Q | 1 |
| Q | 0 | B4K587 | H | 0 | N | 8 | M | 0 | | I | 3 | | H | 5 | A6DTI5 | L | 2 | | Q | 0 | | I | 0 | | T | 2 | | A | 1 |
| K | 0 | Q8I189 | L | 0 | S | 6 | P | 0 | | C | 3 | | F | 4 | A6DQD9 | C | 2 | | F | 0 | | E | 0 | | H | 2 | | F | 0 |
| E | 0 | B3MSR6 | R | 0 | T | 5 | L | 0 | | S | 5 | | C | 4 | A6DR17 | M | 1 | | P | 0 | | H | 0 | | A | 2 | | M | 0 |
| R | 0 | B4I238 | A | 0 | V | 5 | I | 0 | | V | 5 | | S | 3 | G0L5L5 | Y | 1 | | D | 0 | | T | 0 | | C | 1 | | W | 0 |
| L | 0 | Q9VE24 | T | 0 | D | 4 | H | 0 | | M | 5 | | V | 2 | | K | 1 | | C | 0 | | N | 0 | | S | 1 | | S | 0 |
| | | Q8I1I2 | C | 0 | K | 4 | R | 0 | | Q | 5 | | N | 2 | | N | 1 | | R | 0 | | V | 0 | | K | 1 | | Y | 0 |
| | | B3P396 | E | 0 | E | 3 | K | 0 | | T | 5 | | G | 1 | | D | 0 | | I | 0 | | F | 0 | | Q | 0 | | K | 0 |
| | | B4PM30 | D | 0 | F | 2 | F | 0 | | E | 0 | | W | 1 | | G | 0 | | | | | | | | V | 0 | | C | 0 |
| | | B4QTX6 | N | 0 | L | 1 | | | | W | 0 | | T | 1 | | W | 0 | | | | | | | | G | 0 | | P | 0 |
| | | B8M2K9 | | | M | 1 | | | | N | 0 | | R | 0 | | Q | 0 | | | | | | | | D | 0 | | T | 0 |
| | | A3LZV3 | | | I | 0 | | | | K | 0 | | K | 0 | | H | 0 | | | | | | | | I | 0 | | I | 0 |
| | | A6DJ41 | | | G | 0 | | | | R | 0 | | E | 0 | | R | 0 | | | | | | | | R | 0 | | E | 0 |
| | | A6DJ58 | | | W | 0 | | | | P | 0 | | D | 0 | | E | 0 | | | | | | | | P | 0 | | V | 0 |
| | | | | | C | 0 | | | | | | | | | | P | 0 | | | | | | | | E | 0 | | R | 0 |

**(C)**

## Calcium binding 1 consensus

| 5 | | | 6 | | 7 | | 8 | | | 9 | | 10 | | | 11 | | 12 | | 13 | | 14 | | 15 | | 16 | |
|---|---|---|---|---|---|---|---|---|---|---|---|---|---|---|---|---|---|---|---|---|---|---|---|---|---|---|
| | % | | | % | | % | | % | | | % | | % | | | % | | % | | % | | % | | % | | % |
| **P** | **82** | **x(0-1)** | **N** | **89** | **I** | **56** | **L** | **42** | **x(0-16)** | **F** | **24** | **I** | **62** | **x(0-6)** | **L** | **30** | **A** | **33** | **D** | **96** | **D** | **76** | **L** | **37** | **G** | **57** |
| M | 4 | 24 seq. | H | 2 | V | 26 | I | 29 | B3T1C6 | L | 23 | V | 13 | A8HPB7 | M | 25 | T | 16 | E | 1 | Q | 14 | M | 17 | R | 16 |
| Y | 1 | | D | 2 | F | 7 | V | 24 | | V | 18 | L | 12 | | V | 10 | V | 10 | H | 0 | S | 2 | Q | 13 | A | 5 |
| K | 1 | | K | 1 | L | 4 | M | 0 | | I | 16 | F | 6 | | F | 8 | S | 6 | G | 0 | E | 2 | V | 7 | N | 4 |
| R | 1 | | R | 0 | A | 1 | F | 0 | | Y | 10 | M | 1 | | I | 8 | L | 5 | N | 0 | T | 1 | H | 6 | T | 4 |
| L | 1 | | S | 0 | M | 0 | C | 0 | | M | 2 | A | 0 | | T | 3 | I | 5 | R | 0 | N | 0 | I | 4 | D | 4 |
| Q | 0 | | Q | 0 | T | 0 | A | 0 | | W | 2 | Y | 0 | | Y | 3 | G | 4 | Y | 0 | H | 0 | A | 2 | S | 4 |
| T | 0 | | G | 0 | Y | 0 | | | | A | 0 | C | 0 | | W | 2 | M | 4 | | | A | 0 | W | 2 | K | 0 |
| A | 0 | | P | 0 | R | 0 | | | | T | 0 | P | 0 | | A | 1 | C | 3 | | | C | 0 | E | 1 | Q | 0 |
| S | 0 | | A | 0 | | | | | | H | 0 | T | 0 | | D | 1 | F | 2 | | | M | 0 | T | 1 | H | 0 |
| N | 0 | | | | | | | | | N | 0 | | | | H | 0 | P | 2 | | | L | 0 | F | 1 | P | 0 |
| E | 0 | | | | | | | | | S | 0 | | | | N | 0 | W | 1 | | | G | 0 | Y | 1 | I | 0 |
| D | 0 | | | | | | | | | C | 0 | | | | C | 0 | Y | 0 | | | F | 0 | N | 0 | C | 0 |
| V | 0 | | | | | | | | | Q | 0 | | | | Q | 0 | N | 0 | | | | | G | 0 | L | 0 |
| H | 0 | | | | | | | | | | | | | | S | 0 | Q | 0 | | | | | C | 0 | W | 0 |
| G | 0 | | | | | | | | | | | | | | P | 0 | R | 0 | | | | | S | 0 | Y | 0 |
| F | 0 | | | | | | | | | | | | | | E | 0 | | | | | | | R | 0 | F | 0 |
| I | 0 | | | | | | | | | | | | | | G | 0 | | | | | | | D | 0 | E | 0 |
| W | 0 | | | | | | | | | | | | | | | | | | | | | | | | | |

**(D)**

## Calcium binding 2 consensus

| 309 | | 310 | | | 311 | | 312 | | 313 | | 314 | | | 315 | | 316 | | | 317 | | 318 | | 319 | |
|---|---|---|---|---|---|---|---|---|---|---|---|---|---|---|---|---|---|---|---|---|---|---|---|---|
| | % | | % | | | % | | % | | % | | % | | | % | | % | | | % | | % | | % |
| **N** | **72** | **T** | **94** | **x(0-2)** | **I** | **40** | **V** | **43** | **I** | **37** | **F** | **57** | **x(0-1)** | **T** | **42** | **S** | **65** | **x(0-1)** | **D** | **99** | **N** | **61** | **G** | **99** |
| D | 15 | S | 2 | D5EMJ7 | L | 32 | I | 42 | V | 31 | Y | 22 | 188 seq. | S | 12 | T | 12 | E6X913 | E | 0 | H | 32 | A | 0 |
| E | 3 | A | 2 | D5EN67 | V | 9 | L | 8 | F | 10 | V | 7 | | L | 9 | G | 11 | | R | 0 | Q | 5 | S | 0 |
| K | 2 | L | 0 | D5EN14 | Y | 5 | F | 3 | L | 10 | L | 6 | | M | 7 | A | 9 | | T | 0 | S | 0 | R | 0 |
| S | 1 | Q | 0 | D5EN19 | M | 4 | T | 0 | M | 4 | I | 2 | | I | 5 | N | 0 | | Y | 0 | M | 0 | P | 0 |
| R | 1 | I | 0 | D5EN12 | F | 4 | M | 0 | Y | 1 | W | 0 | | V | 5 | D | 0 | | A | 0 | D | 0 | T | 0 |
| H | 0 | R | 0 | D5EMJ6 | W | 1 | A | 0 | W | 1 | R | 0 | | F | 3 | C | 0 | | | | A | 0 | Q | 0 |
| Q | 0 | G | 0 | D5ENX2 | A | 1 | Q | 0 | C | 1 | A | 0 | | A | 3 | P | 0 | | | | P | 0 | N | 0 |
| T | 0 | V | 0 | C0FVD6 | T | 0 | | | A | 0 | M | 0 | | W | 2 | Q | 0 | | | | F | 0 | | |
| G | 0 | N | 0 | F4AN26 | H | 0 | | | H | 0 | H | 0 | | G | 2 | V | 0 | | | | I | 0 | | |
| P | 0 | C | 0 | | C | 0 | | | T | 0 | C | 0 | | C | 2 | H | 0 | | | | | | | |
| I | 0 | P | 0 | | D | 0 | | | Q | 0 | S | 0 | | Y | 1 | I | 0 | | | | | | | |
| Y | 0 | Y | 0 | | R | 0 | | | S | 0 | Q | 0 | | N | 0 | | | | | | | | | |
| W | 0 | | | | S | 0 | | | G | 0 | G | 0 | | Q | 0 | | | | | | | | | |
| | | | | | | | | | | | | | | H | 0 | | | | | | | | | |
| | | | | | | | | | | | | | | E | 0 | | | | | | | | | |
| | | | | | | | | | | | | | | D | 0 | | | | | | | | | |
| | | | | | | | | | | | | | | K | 0 | | | | | | | | | |

# (E)

## New signature

| 200 | % | 201 | % | 202 | % | 203 | % | x(0,1) | 204 | % | x(0,34) |
|---|---|---|---|---|---|---|---|---|---|---|---|
| **P** | **93** | **F** | **75** | **F** | **66** | **L** | **55** | B9NR18 | **Y** | **39** | B2AAG4 |
| N | 2 | W | 12 | L | 9 | M | 12 | B9NR19 | W | 9 | |
| K | 1 | L | 4 | C | 9 | A | 10 | D5EPW8 | V | 8 | |
| S | 0 | I | 2 | M | 5 | V | 6 | B9R4R2 | M | 5 | |
| R | 0 | V | 1 | Y | 3 | I | 5 | | T | 5 | |
| D | 0 | Y | 1 | A | 2 | C | 2 | | F | 4 | |
| A | 0 | M | 0 | V | 0 | G | 1 | | A | 4 | |
| T | 0 | C | 0 | I | 0 | T | 1 | | H | 4 | |
| Q | 0 | T | 0 | S | 0 | S | 1 | | Q | 3 | |
| G | 0 | A | 0 | T | 0 | F | 1 | | S | 3 | |
| H | 0 | H | 0 | H | 0 | Y | 0 | | L | 2 | |
| F | 0 | S | 0 | W | 0 | H | 0 | | N | 2 | |
| | | Q | 0 | | | Q | 0 | | I | 1 | |
| | | G | 0 | | | W | 0 | | K | 1 | |
| | | K | 0 | | | N | 0 | | G | 1 | |
| | | | | | | E | 0 | | C | 0 | |
| | | | | | | | | | E | 0 | |
| | | | | | | | | | R | 0 | |
| | | | | | | | | | D | 0 | |

| 205 | % | 206 | % | x(0,1) | 207 | % | 208 | % | x(0,5) | 209 | % | 210 | % | 211 | % |
|---|---|---|---|---|---|---|---|---|---|---|---|---|---|---|---|
| **L** | **29** | **A** | **30** | D5EPW8 | **F** | **26** | **T** | **20** | A9VAR3 | **A** | **39** | **P** | **62** | **H** | **99** |
| V | 27 | S | 25 | | Y | 17 | N | 12 | | D | 8 | V | 13 | K | 0 |
| I | 9 | G | 11 | | H | 12 | G | 7 | | R | 7 | T | 7 | Y | 0 |
| Y | 9 | N | 10 | | T | 11 | H | 5 | | H | 6 | M | 5 | G | 0 |
| F | 8 | P | 6 | | P | 9 | K | 5 | | E | 5 | I | 2 | N | 0 |
| M | 5 | H | 4 | | L | 8 | Y | 5 | | K | 4 | A | 2 | V | 0 |
| A | 3 | T | 3 | | M | 4 | F | 5 | | L | 4 | C | 1 | R | 0 |
| W | 2 | C | 2 | | S | 3 | V | 4 | | G | 3 | G | 1 | I | 0 |
| C | 1 | E | 2 | | A | 1 | S | 4 | | N | 3 | S | 0 | D | 0 |
| H | 1 | W | 1 | | I | 1 | P | 4 | | S | 3 | L | 0 | | |
| T | 0 | Q | 0 | | Q | 1 | I | 4 | | P | 2 | N | 0 | | |
| S | 0 | D | 0 | | G | 0 | Q | 4 | | T | 2 | H | 0 | | |
| Q | 0 | V | 0 | | W | 0 | A | 3 | | Q | 1 | Q | 0 | | |
| N | 0 | L | 0 | | V | 0 | L | 3 | | V | 1 | E | 0 | | |
| E | 0 | M | 0 | | C | 0 | D | 2 | | I | 1 | F | 0 | | |
| | | F | 0 | | N | 0 | W | 2 | | M | 1 | | | | |
| | | Y | 0 | | E | 0 | R | 1 | | F | 0 | | | | |
| | | | | | D | 0 | M | 0 | | C | 0 | | | | |
| | | | | | | | E | 0 | | Y | 0 | | | | |
| | | | | | | | C | 0 | | W | 0 | | | | |
