# Supplementary material for: Matching the Diversity of Sulfated Biomolecules: Creation of a Classification Database for Sulfatases Reflecting Their Substrate Specificity
Source: PLoS One. 2016 Oct 17;11(10):e0164846. doi: 10.1371/journal.pone.0164846 (PMC5066984; doi:10.1371/journal.pone.0164846)
Supplement: S3 Fig — The alkylsulfodioxygenases consensus sequence was deduced from an alignment of 111 sequences, extracted from the global multi-alignment of 469 dioxygenases. This latter alignment was obtained using the MAFFT program with the L-INS-i algorithm as the iterative refinement method. The consensus sequence appears in bold. The blue numbers indicate the position of amino acids in the reference sequence AtsK (Q9WWU5). For each position, the amino acids present and the percentage of sequence that they represent in the multi-alignment are indicated. The value 0% means that the amino acid is present in less than 1% of sequences. The accession numbers of sequences responsible of insertions in the consensus sequence or their number is indicated at positions "x". (PDF) [file pone.0164846.s003.pdf]

Alkylsulfodioxygenase consensus

156		157		158		159		160		161		162	
	%		%		%		%		%		%		%
**D**	**68**	**N**	**29**	**L**	**100**	**W**	**87**	**A**	**98**	**V**	**54**	**H**	**100**
E	27	T	21			R	10	V	1	L	23		
N	3	K	18			V	1			I	13		
		R	10							R	3		
		Q	6							T	2		
		G	4							E	1		
		S	3							M	0		
		E	1										
		D	1										
		A	1										

163		164			165		166			167		168	
	%		%	**x(0,1)**		%		%	**x(0,2)**		%		%
**T**	**58**	**N**	**99**	A9H456	**A**	**27**	**Y**	**81**	Q10ZT1	**D**	**98**	**Y**	**96**
S	30	F	0	C2AL50	D	24	F	17	C2AL50	E	0	L	1
G	8			D5UT36	E	16	G	0	D5UT36	S	0	Q	0
D	1				R	11	A	0				H	0
N	0				Q	6							
					L	4							
					V	4							
					T	1							
					P	0							
					K	0							
					H	0							
