# Supplementary material for: Matching the Diversity of Sulfated Biomolecules: Creation of a Classification Database for Sulfatases Reflecting Their Substrate Specificity
Source: PLoS One. 2016 Oct 17;11(10):e0164846. doi: 10.1371/journal.pone.0164846 (PMC5066984; doi:10.1371/journal.pone.0164846)
Supplement: S4 Fig — The alkylsulfohydrolases consensus sequence was deduced from an alignment of 370 sequences obtained using the MAFFT program with the L-INS-i algorithm as iterative refinement method. The consensus sequence appears in bold. The blue numbers indicate the position of amino acids in the reference sequence SdsA1 (Q9I5I9). Amino acids involved in binding sulfate are shown in red in the consensus sequences. For each position, the amino acids present and the percentage of sequence that they represent in the multi-alignment are indicated. The value 0% means that the amino acid is present in less than 1% of sequences. (PDF) [file pone.0164846.s004.pdf]

# Alkylsulfohydrolase consensus

| 298 | % | 299 | % | 300 | % | 301 | % | 302 | % | 303 | % | 304 | % | 305 | % | 306 | % | 307 | % |
|---|---|---|---|---|---|---|---|---|---|---|---|---|---|---|---|---|---|---|---|
| **A** | **80** | **E** | **84** | **N** | **57** | **A** | **37** | **T** | **51** | **H** | **53** | **T** | **48** | **L** | **51** | **H** | **84** | **N** | **98** |
| G | 16 | D | 12 | D | 16 | T | 16 | Y | 17 | G | 18 | N | 16 | M | 23 | P | 15 | D | 0 |
| S | 1 | N | 2 | L | 13 | V | 14 | V | 11 | Q | 6 | G | 14 | F | 11 | | | Q | 0 |
| I | 1 | A | 0 | V | 5 | F | 7 | N | 5 | R | 5 | S | 8 | Q | 4 | | | A | 0 |
| T | 0 | | | T | 2 | I | 5 | S | 5 | A | 4 | C | 3 | I | 4 | | | | |
| | | | | E | 1 | C | 5 | Q | 2 | K | 3 | H | 3 | W | 2 | | | | |
| | | | | C | 1 | M | 4 | F | 2 | D | 3 | A | 1 | V | 1 | | | | |
| | | | | I | 1 | L | 3 | I | 1 | N | 2 | V | 1 | Y | 0 | | | | |
| | | | | S | 0 | Y | 3 | L | 0 | S | 0 | L | 1 | | | | | | |
| | | | | M | 0 | S | 2 | A | 0 | V | 0 | I | 0 | | | | | | |
| | | | | | | D | 0 | M | 0 | C | 0 | Q | 0 | | | | | | |
| | | | | | | W | 0 | P | 0 | P | 0 | | | | | | | | |
| | | | | | | G | 0 | C | 0 | W | 0 | | | | | | | | |
| | | | | | | | | G | 0 | E | 0 | | | | | | | | |
| | | | | | | | | | | T | 0 | | | | | | | | |

| 308 | % | 309 | % | 310 | % | 311 | % | 312 | % | 313 | % | 314 | % | 315 | % | 316 | % | 317 | % |
|---|---|---|---|---|---|---|---|---|---|---|---|---|---|---|---|---|---|---|---|
| **L** | **42** | **Y** | **61** | **T** | **81** | **L** | **73** | **R** | **99** | **G** | **99** | **A** | **83** | **K** | **41** | **V** | **54** | **R** | **99** |
| I | 26 | L | 24 | A | 7 | I | 13 | C | 0 | | | T | 9 | Q | 16 | T | 18 | G | 0 |
| F | 13 | Q | 3 | S | 7 | P | 5 | T | 0 | | | D | 2 | L | 14 | I | 11 | S | 0 |
| V | 10 | H | 2 | P | 4 | F | 5 | M | 0 | | | S | 1 | E | 10 | Y | 9 | | |
| T | 6 | I | 2 | D | 0 | M | 1 | | | | | G | 1 | P | 4 | F | 1 | | |
| P | 0 | S | 1 | | | V | 0 | | | | | L | 0 | R | 3 | P | 1 | | |
| | | V | 1 | | | | | | | | | V | 0 | V | 2 | A | 1 | | |
| | | M | 0 | | | | | | | | | E | 0 | A | 1 | N | 0 | | |
| | | A | 0 | | | | | | | | | | | D | 1 | L | 0 | | |
| | | G | 0 | | | | | | | | | | | M | 0 | S | 0 | | |
| | | T | 0 | | | | | | | | | | | S | 0 | M | 0 | | |
| | | E | 0 | | | | | | | | | | | H | 0 | H | 0 | | |
| | | C | 0 | | | | | | | | | | | Y | 0 | | | | |
| | | | | | | | | | | | | | | F | 0 | | | | |
