# Supplementary figures and images for: Matching the Diversity of Sulfated Biomolecules: Creation of a Classification Database for Sulfatases Reflecting Their Substrate Specificity
Source: PLoS One. 2016 Oct 17;11(10):e0164846. doi: 10.1371/journal.pone.0164846 (PMC5066984; doi:10.1371/journal.pone.0164846)

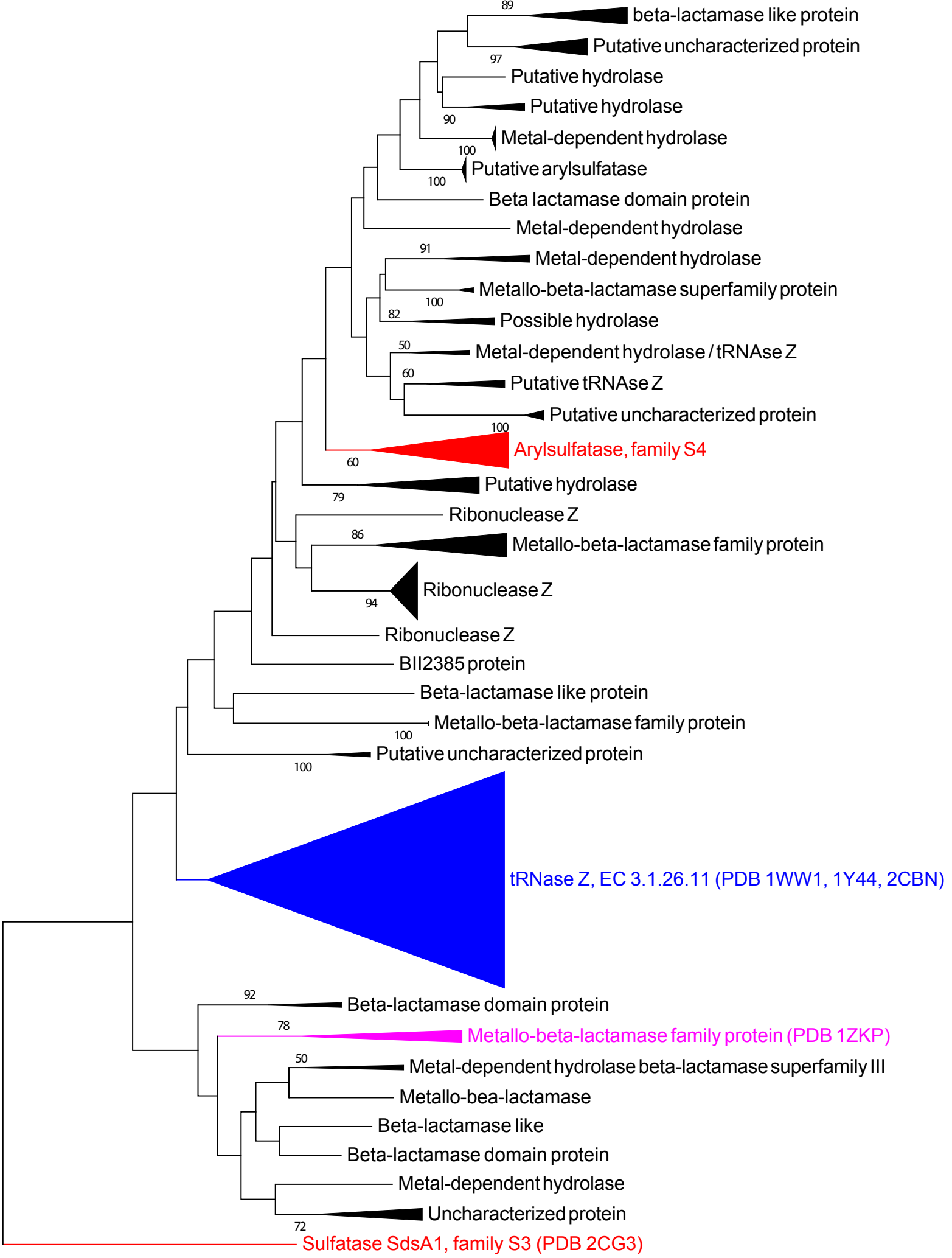

0.2

Supplement: S5 Fig — The tree was obtained by maximum likelihood with RAxML using the substitution matrix WAG from 187 positions from an alignment of 240 sequences. The blue clade contains the characterized tRNases Z (EC 3.1.26.11) and related sequences. The black clades and the isolated sequences (not supported by high bootstrap values) contain no biochemically-characterized enzymes. The family S4 of the sulfatases is shown in red. All the resolved three-dimensional structures are indicated. Only bootstrap values above 50% are shown. The sequence belonging to the S3 family of sulfatases Q9I5I9 (SdsA1 from Pseudomonas aeruginosa PAO1) was used as an outgroup. (PDF) [file pone.0164846.s005.pdf]
