# Supplementary material for: Matching the Diversity of Sulfated Biomolecules: Creation of a Classification Database for Sulfatases Reflecting Their Substrate Specificity
Source: PLoS One. 2016 Oct 17;11(10):e0164846. doi: 10.1371/journal.pone.0164846 (PMC5066984; doi:10.1371/journal.pone.0164846)
Supplement: S2 Fig — The tree was obtained by maximum likelihood with RaxML using MTMAMF as a substitution matrix from an alignment of 4005 sequences and 329 positions. The clades represent the subfamilies according the proposed nomenclature. For the subfamilies with a characterized activity, the activity name and the corresponding EC number are indicated. All resolved three-dimensional structures are indicated. Orphean sequences (which were not include in a clade) are annotated S1_N.C. (for non-classified). Only bootstrap values above 60% are shown. (PDF) [file pone.0164846.s002.pdf]

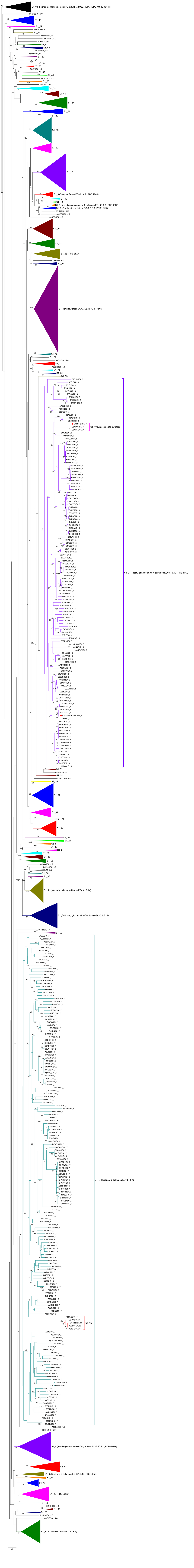
S1_0 (Phophonate monoesterase ; PDB 2VQR, 2W8S, 4UPI, 4UPL, 4UPK, 4UPH)
S1_46
S1_59
S1_57
S1_67
S1_63
S1_52
S1_54
S1_60
S1_55
S1_56
S1_68
S1_58
S1_53
S1_61
S1_64
S1_24
S1_69
S1_15
S1_14
S1_13
S1_3 (Steryl-sulfatase EC=3.1.6.2 ; PDB 1P49)
S1_47
S1_42
S1_5 (N-acetylgalactosamine-6-sulfatase EC=3.1.6.4 ; PDB 4FDI)
S1_1 (Cerebroside sulfatase EC=3.1.6.8 ; PDB 1AUK)
S1_20
S1_17
S1_23 ; PDB 3ED4
S1_22
S1_4 (Arylsulfatase EC=3.1.6.1 ; PDB 1HDH)
S1_50
S1_62
S1_18
S1_71
S1_31
S1_33
S1_10 (Glucosinolate sulfatase)
S1_2 (N-acetylgalactosamine-4-sulfatase EC=3.1.6.12 ; PDB 1FSU)
S1_32
S1_32
S1_38
S1_16
S1_19
S1_40
S1_44
S1_70
S1_29
S1_51
S1_49
S1_21
S1_26
S1_28
S1_25
S1_34
S1_36
S1_35
S1_11 (Mucin-desulfating sulfatase EC=3.1.6.14)
S1_6 (N-acetylglucosamine-6-sulfatase EC=3.1.6.14)
S1_72
S1_7 (Iduronate-2-sulfatase EC=3.1.6.13)
S1_66
S1_8 (N-sulfoglucosamine sulfohydrolase EC=3.10.1.1 ; PDB 4MHX)
S1_48
S1_9 (Iduronate-2-sulfatase EC=3.1.6.13 ; PDB 3B5Q)
S1_65
S1_43
S1_27 ; PDB 2QZU
S1_30
S1_45
S1_37
S1_12 (Choline sulfatase EC=3.1.6.6)
0.5
